# Using pictographs as traits to explore morphological diversity in sharks

**DOI:** 10.1002/ece3.9761

**Published:** 2023-01-24

**Authors:** Zachary A. Siders, Fabio P. Caltabellotta, Katherine B. Loesser, Lauren B. Trotta, Benjamin Baiser

**Affiliations:** ^1^ Fisheries and Aquatic Sciences Program, School of Forest, Fisheries, and Geomatic Sciences University of Florida Florida Gainesville USA; ^2^ Coastal and Marine Laboratory Florida State University St. Teresa Florida USA; ^3^ Department of Oceanography and Coastal Sciences Louisiana State University Baton Rouge Louisiana USA; ^4^ Department of Wildlife Ecology and Conservation University of Florida Gainesville Florida USA

**Keywords:** ancestral trait reconstruction, digitization, elasmobranchs, geometric morphometrics

## Abstract

Body shape is a foundational trait on the differences between species. However, morphological measurements can be simplifying and, for many taxa, can be distorted upon preservation or are difficult to collect due to a species' habit or size. Scientific illustrations, or pictographs, provide information on a species' morphology but are rarely used as traits. Here, we demonstrate the use of pictographs using two shark clades: Lamniformes and Carcharhinidae + Sphyrnidae. After collecting 473 pictographs from 67 species across 12 sources, we used landmarking to show that measurements derived from pictographs do not substantially differ from those garnered from specimens. We then used Elliptical Fourier Analysis and principal components analysis to construct a multivariate morphospace. Using global shape measurements, we evaluated whether substantial variability in body shape was introduced by habitat association, endemism, or illustrator. We found that a species' habitat preference strongly influenced the discovery rate of pictographs and the within‐species similarity. While illustrations varied within a species, only a limited set of illustrators exhibited significant systematic variability. We also demonstrated the utility of pictographs in two common applications. For ancestral trait reconstruction, we developed a simple extension to estimate body shapes from principal components and, in doing so, observed that the Lamnid body plan diverged from the rest of Lamniformes ~100 MYA. For phylogenetic generalized linear mixed models (PGLMM), we found that the pictographs had greater explanatory power than traditional morphological measurements. We used the PGLMM to show that higher endemism across Carcharhinidae + Sphyrnidae taxa correlates with body shapes that have caudal fins with small heterocercal angles and more pronounced second dorsal/anal fins. We concluded that pictographs are likely an undervalued and easy‐to‐digitize data source on a species' body shape with numerous established methods for comparing pictographs and assessing variability.

## Graphical abstract

Scientific illustrations are useful traits that should be included in trait‐based studies. We show there similarity to specimen‐based measurements and find little systematic variability across illustrators. We highlight their utility in enhancing two common trait‐based analyses: ancestral trait reconstruction and phylogenetic generalized linear mixed models.

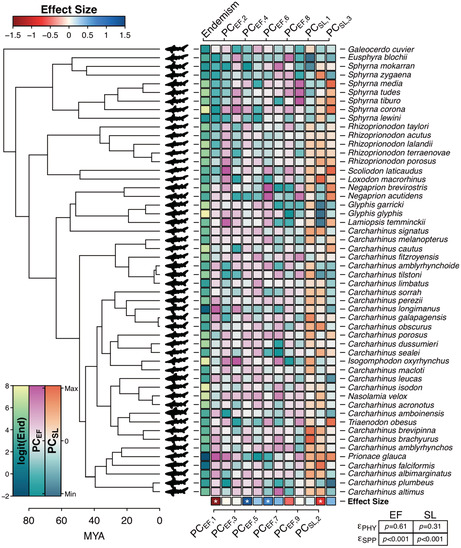

## 1 INTRODUCTION

Species' traits are broadly used to understand the intra‐ and interspecific relationships between organisms across ecology (Cadotte et al., 2011; McGill et al., 2006; Moen, 2019). Often, traits related to the species' morphology are of interest and come from multiple measures of morphology from specimens in or ex situ or, recently, from photogrammetry or tomography (Gutiérrez et al., 2018). These approaches can capture multiple facets of an organism's morphology and, through multivariate analyses, be decomposed into major axes of variation (Adams et al., 2013). However, for some species, in situ measurements may be difficult to obtain, ex situ specimens may be rare, distorted, or damaged, and species may be difficult or impossible to photograph or scan (though see Moyer et al., 2021). These issues are common in rare species, megafauna, and even more common in organisms lacking calcified and durable body parts; all qualities common to marine organisms.

Pictographs, a two‐dimensional representation of a three‐dimensional object, may provide an alternative to the use of specimens in the generation of morphometric traits (Sternes et al., 2022; Sternes & Shimada, 2020). Typically available as scientific illustrations, these graphical data may offer several advantages to morphological measurements: (1) pictographs are readily accessible in field guides, plates, and species descriptions; (2) even rare taxa often have at least one pictograph; and (3) pictographs can be scanned quickly compared to taking measurements, and decomposed similarly to photogrammetric or tomographic data (Adams et al., 2013; Palci & Lee, 2019). Furthermore, pictographs typically attempt to represent the species as they would be encountered in situ and can be a holistic rendering of the morphology that morphological measurements may miss (Rohlf, 1986). Together, these advantages may circumvent some of the specimen availability issues as well as, potentially, the distortions that can result from specimen preservation (Jones & Geen, 1977).

However, scientific illustrations are an interpretation of a species' morphology and can vary considerably. This variability can result from the type of specimen used, the skill of the artist, the perspective of the pictograph, and the goal of the rendering. Additionally, specimen availability and distortions can induce uneven variability across the species pool that can propagate in downstream analyses. Hyper‐rare species may have only one or two specimens available to illustrate, or their pictographs may occur in only a few sources resulting in low variability. The “commonness” of circumglobal species might result in the opposite phenomenon where their frequency of illustration increases the variability. In rare, uncommon, or species with narrow distributions, low variability may result from fewer illustrations that are consistent from fewer specimens or high variability may result from discrepancies across the few specimens. One solution is to use a single encompassing source (as in Sternes et al., 2022; Sternes & Shimada, 2020), but this makes an implicit assumption that this source's illustrations are representative of the species' morphology and that the illustrator's artistic license is applied uniformly across species. Unfortunately, there is no objective truth to an artistic rendering so it is prudent to use multiple sources of pictographs and to assess the variation across them.

With their potential to be a rich data source on a species' morphology, methods are needed for processing pictographs into morphological traits and for assessing the systematic variation that may result from which pictographs are included. For the former, the subfield of geometric morphometrics, “a statistical analysis of form” (sensu Mitteroecker & Gunz, 2009), can be used to analyze landmarks, either specific loci or Cartesian coordinates (Baken et al., 2021; Mitteroecker & Gunz, 2009; Rohlf, 1986). Common throughout comparative morphology studies (Adams et al., 2013), geometric morphometrics can serve as an alternative to discrete qualitative traits about shape by generating continuous quantitative traits. These continuous traits have the potential to enumerate macro‐ and micromorphological features of the species represented for use in a variety of analyses (Baken et al., 2021; Palci & Lee, 2019). Reconstructive techniques, such as Fourier analysis (Lestrel, 1982; Rohlf, 1986), can be used in lieu of defining specific loci to approximate the entire pictograph as a series of sine and cosine waves. Additionally, advancements in open‐access software have facilitated a broader application of geometric morphometrics to photogrammetric, tomographic, and, even, standard photographs datasets (Baken et al., 2021; Emmons et al., 2019).

Here, we outline the methods needed to capture and quantitatively analyze pictographs. Our objectives were to (1) identify major sources of systematic variation; (2) compare the continuous trait values resulting from multivariate decomposition of the pictographs to those produced from common morphological measurements; and (3) define some of the challenges for using pictographs. To accomplish our objectives, we collected a set of pictographs for two lineages of sharks, the sister families of Carcharinidae and Sphyrnidae, and the order Lamniformes, from multiple illustrators. Sharks make particularly strong candidates for the use of pictographs as their cartilage can be distorted through preservation (Irschick et al., 2017; Jones & Geen, 1977), the generally large size of taxa limits the application of tomography, and there is a lack of readily available whole specimens (Irschick et al., 2017). Additionally, sharks, while sharing a general body plan, have a wide range of morphological diversity (Sternes & Shimada, 2020; Thomson & Simanek, 1977) that can reduce the descriptive power of common morphological measurements across species.

## 2 MATERIALS AND METHODS

Our methodology followed several steps: (1) data collection and compilation, (2) cleaning and standardization, (3) calculating per species and overall metrics of trait morphospace, (4) assessing sources of variation from attributes (i.e., artist and species), and (5) applying pictographs as morphological traits in ecological and evolutionary contexts.

### 2.1 Species of interest

We chose to focus on shark species from the families Carcharhinidae and Sphyrnidae and the order Lamniformes. These groups represent disparate evolutionary histories making them strong candidates for comparing the use of pictographs as morphological traits (Sorenson et al., 2014; Stein et al., 2018). Carcharhinidae and Sphyrnidae contain 69 described extant species with short divergence times between sister clades relative to the order Lamniformes with only 15 extant species (Sorenson et al., 2014). For both groups, the majority of species are commonly encountered while a minority are rare or hyper‐rare taxa with limited morphological measurements available. We used the time‐dated multigene phylogeny produced by Sorenson et al. (2014) to define the species list and for subsequent analyses. We subset the overall elasmobranch tree into our two clades of interest: Carcharhinidae + Sphyrnidae and Lamniformes.

### 2.2 Pictograph collection and processing

We compiled pictographs of shark lateral profiles from book compendiums or posters either for a specific region (e.g., Requins de Méditerranée et d'Atlantique) or for the globe (e.g., Sharks of the World) available in the literature since 1980. For each reference, pictographs were digitized with a variety of techniques, which included scanning and camera capture, typically using a mobile device (Figure S1a). Each pictograph then underwent digital processing to remove any background, flatten the image to prevent any distortion during capture, select the outline of the pictograph, and transform the outline into a silhouette with Adobe CC Photoshop (Version 20.0.6, Adobe, San Jose, CA, USA, https://www.adobe.com/products/photoshop.html). Next, pictograph silhouettes were read into *R* using the *jpeg* package (Urbanek, 2019; Figure S1b). With the full pictograph set, we wanted to map the relationship between the number of species with pictographs as a function of additional pictograph sources. We tabulated the frequency of species with a given number of illustrators and modeled the species discovery curve in the *sprex* package (Archer, 2016). We calculated the number of observed species using the Chao1 metric (Colwell et al., 2012) and calculated the maximum number of possible illustrations by multiplying the number of species by the number of illustrations (i.e., complete coverage).

A major challenge of using quantitative reconstruction techniques is to properly align the pictographs (Mitteroecker & Gunz, 2009). We chose to use a landmarking technique where common features (e.g., tips of the fins and tip of the rostrum) across the pictographs are identified and used to align the pictographs (Adams et al., 2013). We added these landmarks to each silhouette using the *Momocs* package in *R* (Bonhomme et al., 2014; Figure S1c). We optimized the minimum number of landmarks needed for sufficient alignment using a subset of the most visually dissimilar silhouettes and used this number of landmarks to align all silhouettes. To align the silhouettes, each pictograph was translated to ensure alignment at the apical point of the rostrum landmark and then a Procrustes alignment with a tolerance of 1 × 10^−5^ was used to complete the full alignment (i.e., generalized Procrustes analysis; Adams et al., 2013; Figure S1c).

Each pictograph silhouette was then quantitatively approximated using Elliptical Fourier Analysis (EFA) (Lestrel, 1982; Rohlf, 1986; Figure S1d). This approach reconstructs the outline of each pictograph as a series of sine and cosine waves and renders a set of Elliptical Fourier (EF) coefficients. The number of harmonics to include in EFA was calibrated with a cutoff of 99% cumulative sum harmonic power, and an arbitrary value of 50 was chosen as the maximum possible number of harmonics after inspecting the harmonic power curve. The EF coefficients were then decomposed with principal components analysis (PCA) to identify major axes of variation. We determined the number of significant EF principal components (PC_EF_) in these PCAs using the *PCAtest* package (Camargo, 2022) and used only the significant PCs in subsequent analyses.

### 2.3 Trait morphospace

As there was no way to define a “true” shark pictograph, we chose to identify the morphospace through a variety of metrics. From the pictographs, we derived multiple measures of the morphospace that separated into two categories: per pictograph metrics and overall metrics. For the per pictograph metrics, three common global shape measures (Rosin, 2005) were calculated: (1) Eigen value‐based eccentricity (e.g., elongatedness) describing the aspect ratio (width: length) of the shape, (2) Haralick's circularity (e.g., compactness) describing the inverse coefficient of variation of the radius, and (3) convexity describing the ratio of a pictograph's area to its convex hull's area. These measures allowed comparison across a set of pictographs from the same species.

From the Elliptical Fourier coefficients, we calculated a dissimilarity matrix (Euclidean distance) between all pictographs for a given species resulting in a dissimilarity matrix for each species. We then calculated the part‐worth of each pictograph to the total dissimilarity by dividing the dissimilarity of a given pictograph by the total dissimilarity by species. This provided a ratio of a given pictograph's dissimilarity to the total species dissimilarity ranging from zero to one. From this ratio, we could determine whether a given pictograph was similar (low ratio) or dissimilar (high ratio) to the majority of pictographs for a given species.

### 2.4 Pictograph validation

**FIGURE 1:**
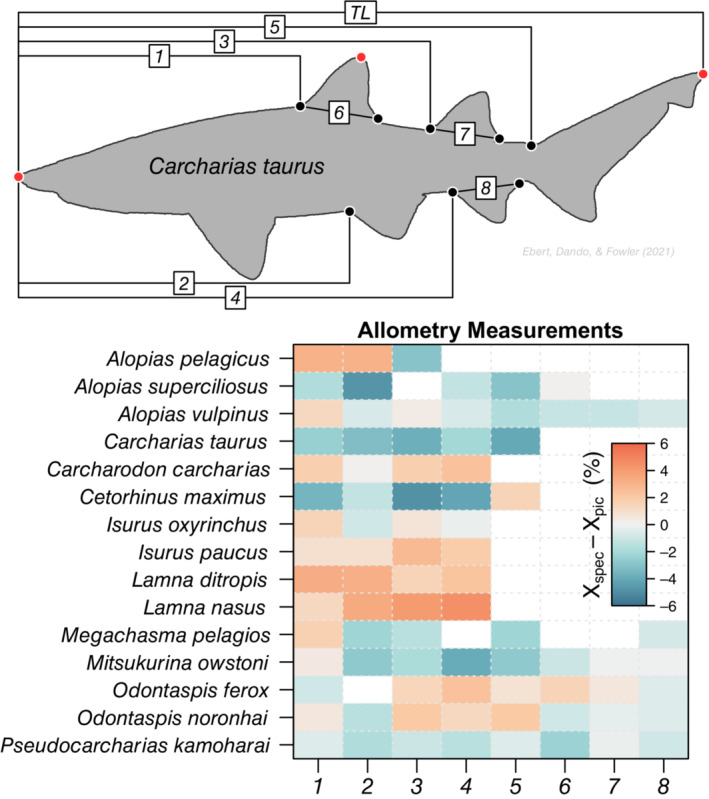
Set of standardized length measurements (Compagno, 1984a) (1–8), we made on each pictograph, shown on an example pictograph of *Carcharias taurus* from (Ebert et al., 2021) (top). For the Lamniform species, the mean residual between the standardized lengths collated from measurements of specimens $\left(X_{\text{spec}}\right)$ in the literature to those measured from the respective set of pictographs $\left(X_{\mathrm{pic}}\right)$ in units of percent of total length (TL). Cooler colors indicate negative residuals $\left(X_{\text{spec}}<X_{\mathrm{pic}}\right)\;$and warmer colors indicate positive residuals $\left(X_{\text{spec}}>X_{\mathrm{pic}}\right)$.

To externally validate whether the pictographs we collected were representative of actual specimens, we collated sets of standardized length measurements (Compagno, 1984a) of Lamniformes species from the literature (Figure 1). Most of these species are circumglobal, which we assumed would increase the odds of finding relevant measurements. We collated measurements of individual or multiple specimens, from multiple sources, and for adults when possible (Data S1). For species with multiple specimens, we took the average and, for sources which only provided a range, we took the midpoint. While many different measurements were possible, we initially chose those that could be feasibly replicated on the pictographs and then those measurements with at least 3 of the 15 Lamniform species represented. Where necessary, we converted length measurements to proportions by dividing by the specimen's total length.

From the set of standardized length measurements, we replicated these measurements on every pictograph we collected (Figure 1). As we were working with pictographs and not actual specimens, we were forced to make some subjective assumptions about where the anterior or posterior point was on a fin. For simplicity and repeatability, but at the cost of some realism, we opted to assume these endpoints occurred at the abrupt angle change when moving from the trunk to a fin along the silhouette's edge (Figure 1). Only pictographs with a clear angle change at each landmark were included in analyses using these standardized lengths. These choices are inherent to using pictographs in lieu of specimens (Sternes & Shimada, 2020) and, thus, useful to determine whether systematic measurement error occurs as a result. We converted the pixel measurements to proportions by dividing by the pictograph's total pixel length to generate measures of proportion of total length. For the Lamniformes pictographs, we subtracted the pictograph‐derived standardized length measurements from the specimen‐derived measurements. We then averaged across the pictograph measurements for each species to get an average residual.

### 2.5 Pictograph assessment

#### 2.5.1 Assessment of variability from habitat preference

We wanted to quantify how the availability of a given species for illustration impacted the number of pictographs and the magnitude of the variability. We chose two metrics as proxies for availability: habitat association and endemism, as there is no global set of rarity measurements for sharks. We extracted habitat association (neritic, pelagic, inland, benthic, nursery, epipelagic, mesopelagic, and bathypelagic) from the IUCN Red List (IUCN, 2015) for each species (see Table S1 for classification scheme) and coded each as a binary variable. For endemism, we calculated a standardized range coverage for each species as one minus the fraction of the ocean's surface area (from the ETOPO1 dataset; Amante, 2009) covered by the IUCN range map (IUCN, 2015) using the *sf* package (Pebesma, 2018). We then regressed the total number of pictographs, the standard deviation in the global shape measures (i.e., eccentricity, circularity, and convexity), and the standard deviation in the EF dissimilarity ratio for each species across the species' pictograph set against these two availability proxies using the *glmmTMB* package (Brooks et al., 2017). This allowed us to assess whether individual habitat associations or a species' distributional range influenced the number of pictographs, the variability in the global shape measures, or the variability in the EF dissimilarity ratio. We used generalized linear models with a Poisson distribution for the total number of pictographs and a log‐normal distribution for the standard deviation in the global shape metrics and in the EF dissimilarity ratio, as the latter is bounded above zero.

#### 2.5.2 Assessment of illustrator variability

We also wanted to quantify the degree to which illustrators exhibited systematic variability across all species and assessed this by modeling the effect of illustrator on a given pictograph's set of global shape measurements and EF dissimilarity ratio. We used generalized linear mixed models with each pictograph's global shape measurements or the EF dissimilarity ratio as the response variables and fixed effects for each illustrator and random effects for source (Equations 1 and 2). We controlled for the within‐species variability by fitting a separate scale parameter for each species (Equations 1 and 2).

(1) $${\hat{Y}}_{i}=\beta_{\text{Ills},i}+\beta_{\text{Source},i,j}+\varepsilon_{i,k}$$

(2) $$\beta_{\text{Source},i,j}\sim \text{Normal}\;\left(0,\sigma_{\beta_{\text{Source}}}\right)$$

where $Y_{i}$ is a given response variable for pictograph i, $\beta_{\text{Ills},i}$ is the fixed effect illustrator, $\beta_{\text{Source},i,j}$ is the random effect for source j, $\sigma_{\beta_{\text{Source}}}$ is the hyper standard deviation of the source random effect, and $\varepsilon_{i,k}$ is the residuals for pictograph i and species k that come from a Gaussian distribution for the global shape measurements (with scale $\sigma_{k}$) and from a Beta distribution for EF dissimilarity ratio (with scale $\phi_{k}$). We used the *emmeans* package to calculate the expected marginal mean effect for each illustrator and assess pairwise differences between illustrators using a Bonferroni correction to adjust the *p*‐values for the multiple comparisons. We also visualized the $\sigma_{k}^{2}$ values estimated in the GLMMs to understand the species variability for the global shape measurements. Low $\sigma_{k}^{2}$ values indicate that all pictographs in a set for the species were relatively similar on a given global shape measurement.

### 2.6 Application examples

We generated two examples to demonstrate the utility of pictographs as traits for two common applications: ancestral trait reconstruction and phylogenetic generalized linear mixed models (PGLMM) (Ives & Helmus, 2011; Li & Ives, 2017). For these applications, we chose to emphasize the added utility of using pictographic traits rather than focusing on the intricacies of the applications' analysis. To demonstrate pictographs in an ancestral trait reconstruction, we used the Lamniform species and corresponding subset of the phylogeny. We first used maximum likelihood to separately estimate the scores from the first and second PC_EF_ axes for each node and across the branches of the phylogeny using continuous mapping (Revell, 2012).

We then extended the *Momocs* package's utility by generating hypothetical silhouettes across the morphospace to visualize lateral profiles estimated by our ancestral state reconstructions. To do this, we built a grid of lateral profiles for the range of the first and second PC_EF_ axes with 100 rows and 100 columns (10,000 profiles total). For a given ancestral trait set of scores from the first and second PC_EF_ axes, we calculated the minimal distance between the set and the grid coordinates to identify the corresponding lateral profile. We performed the same procedure for extant species but used the centroid of each species' scores from the first and second PC_EF_ axes.

For the PGLMM application, we compared two models of endemism for Carcharhinidae and Sphyrnidae. The first model used the lateral profile traits captured by the PC_EF_ scores for each species from the significant PC_EF_ axes. For the second model, we used the standardized length measurements (proportion of total length) of the pictographs. We performed a PCA in the same manner as above on these standardized length measurements (PC_SL_), but normalized the raw measurements using the *bestNormalize* package first (Peterson, 2021). We then calculated the median PC_SL_ score for each species from all the pictographs and used only the scores from significant PC_SL_ axes. We logistically transformed our endemism metric $\left(1-\frac{A_{\text{dist}}}{A_{\text{ocean}}}\right)$ and then scaled and centered all covariates. We used the *phyr* (Li et al., 2020) package to estimate the effects of the PGLMMs with an unconstrained random effect for species and a phylogenetically constrained random effect for species. To calculate the *p*‐value $\left(\alpha =0.05\right)$ for the random effects, we ran additional models with only the unconstrained species random effects and with no species random effects. We used a likelihood ratio test to assess the random effects' significance and compare the PC_EF_ and PC_SL_ models; assuming the difference between −2Δlog‐likelihood (*LL*) of the models came from a one‐sided *χ* ^2^ distribution with one degree of freedom. We also assessed the model fit, *R* ^2^, by calculating the sum of residuals squared and dividing by the total sum of squares. We chose to report the fixed effects for the PC_EF_ and PC_SL_ models with both species' random effects alongside the *p*‐values for those random effects.

## 3 RESULTS

### 3.1 Pictographs collection

We collected 473 pictographs of 67 species across 12 sources with nine unique illustrators with 133 pictographs coming from Lamniformes and 340 pictographs coming from Carcharhinidae + Sphyrnidae (Table S2; Figure S2). Marc Dando, the illustrator for two “Sharks of the World” sources, illustrated the most pictographs in our species pool followed by Emanuela D'Antoni, a scientific illustrator for the Food and Agricultural Organization (Table S3). Visually, we identified some reprints (e.g., Food and Agricultural Organization sources) and discarded two additional sources because they contained no unique pictographs. Unlike typical discovery curves, the expected number of species was known from the outset and, thus, reflects the necessary number of pictographs to achieve at least one pictograph of every species, roughly 250 pictographs (Figure S3). Three landmarks—located at the apical point of the rostrum, the first dorsal fin, and the upper caudal fin lobe—were required to properly align the pictographs (Figure S4). The resulting 2.2 million coordinates were decomposed into 25,000 Fourier coefficients using 15 EFA harmonics (Figure S5).

**FIGURE 2:**
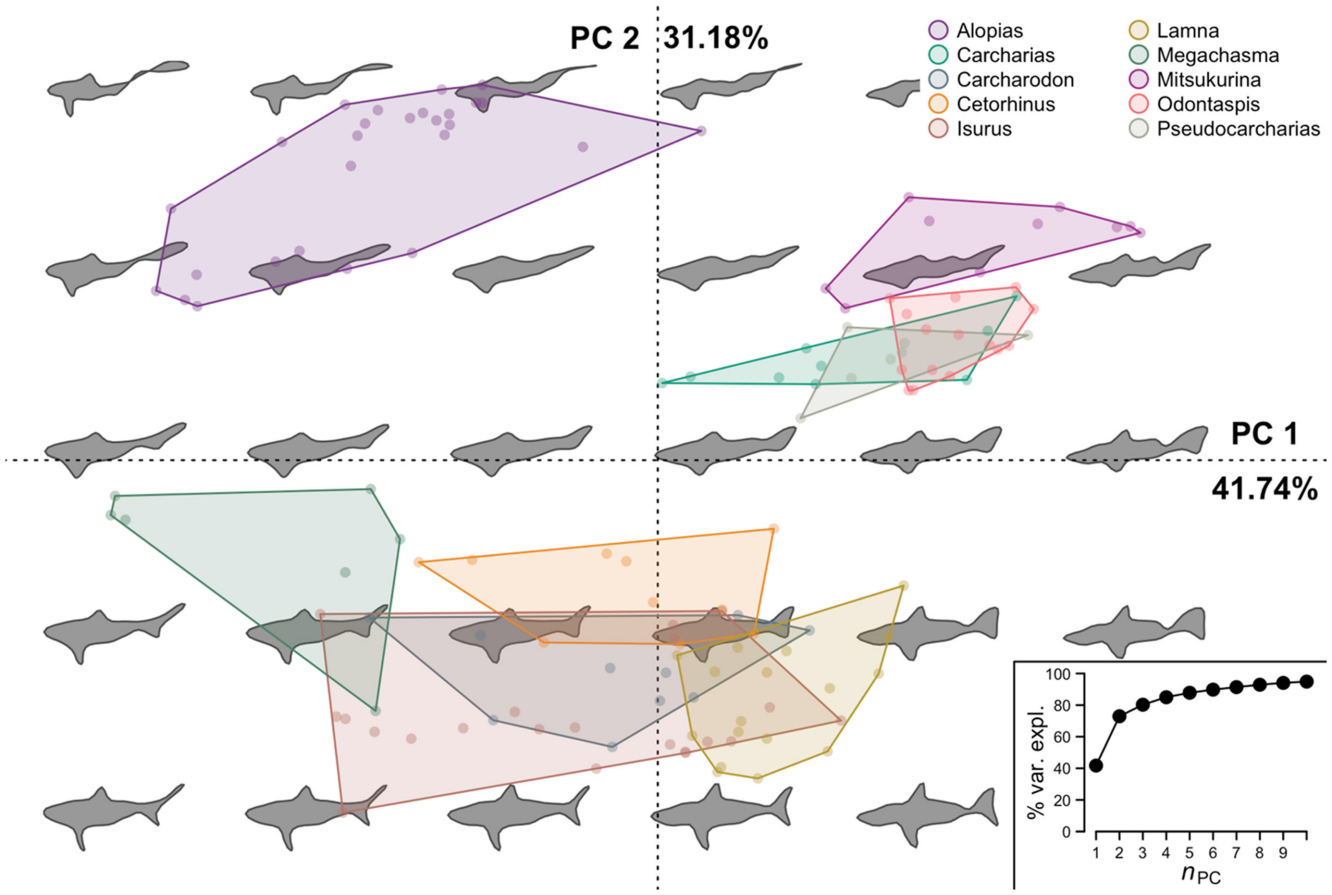
First and second principal components of the Lamniformes lateral profile pictographs using Elliptical Fourier coefficients (PC_EF_). The expected lateral profile is indicated by the silhouettes while the PC_EF_ scores for the individual pictographs are shown with dots and bounded by a convex hull, colored by genera. The percent variance explained of the two PC_EF_ axes is labeled on the *x* and *y* axes and the cumulative percent variance explained of the first ten PC_EF_ is provided in the lower right.

**FIGURE 3:**
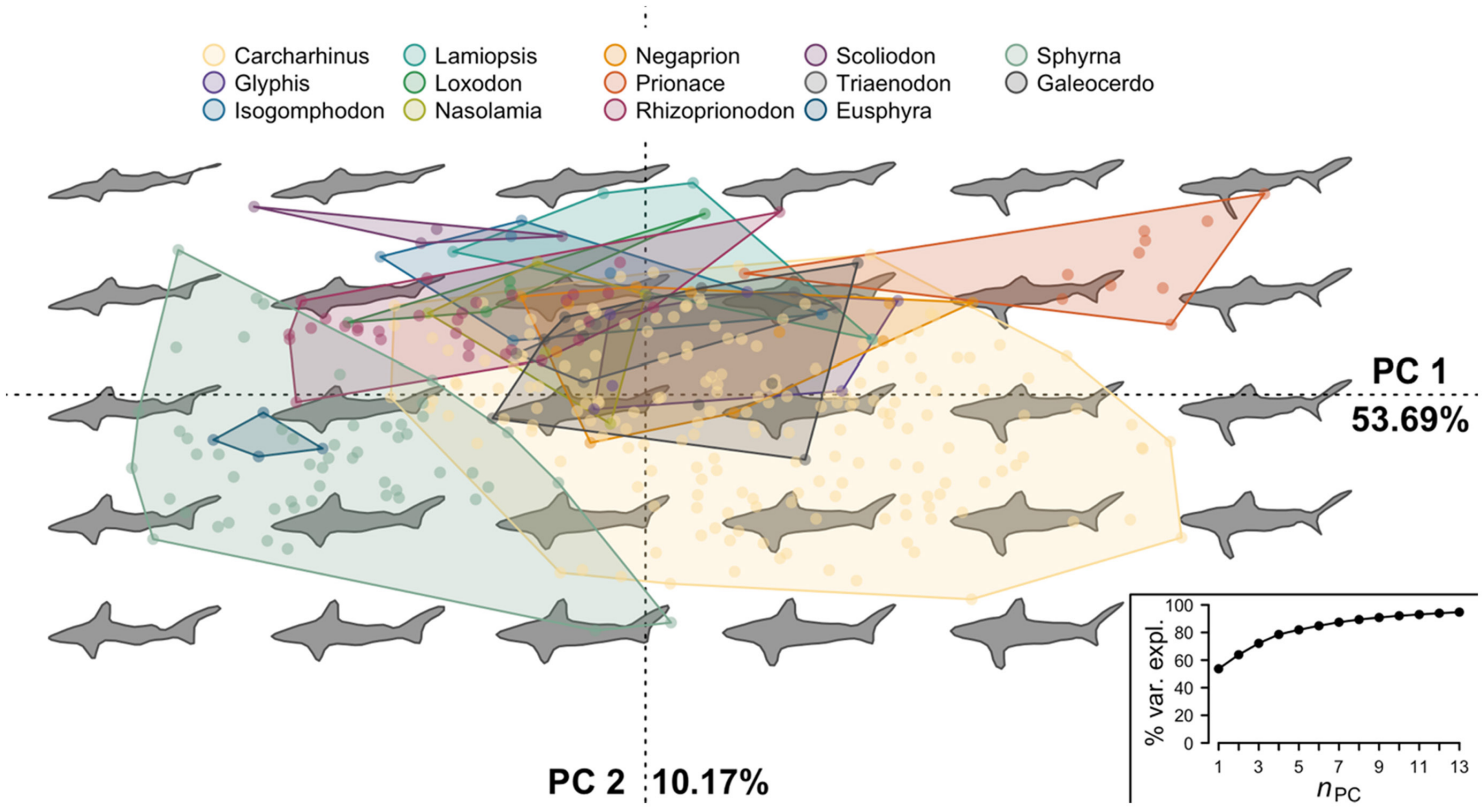
First and second principal components of the Carcharhinidae and Sphyrnidae lateral profile pictographs using Elliptical Fourier coefficients (PC_EF_). The expected lateral profile is indicated by the silhouettes while the PC_EF_ scores for the individual pictographs are shown as dots and bounded by a convex hull, colored by genera. The percent variance explained of the two PC_EF_ axes is labeled on the *x* and y axes and the cumulative percent variance explained of the first 13 PC_EF_ is provided in the lower right.

Lamniformes had seven significant PCs with PC1_EF_ explaining 41.74% and PC2_EF_ explaining 31.18% of the variation (Figure 2). The first two PCs for Lamniformes separated Alopiidae from two other clusters: one of species in the genera Carcharias, Mitsukurina, Odontaspis, and Pseudocarcharias and another of the species in the genera Carcharodon, Cetorhinus, Isurus, Lamna, and Megachasma (Figure 2). The least eccentric (most elongated) shapes had positive PC2_EF_ scores (Figure S6a) while the least convex shapes had negative PC1_EF_ and positive PC2_EF_ scores while (Figure S6b). Carcharinids and Sphyrnids had nine significant PCs in the PCA of the Elliptical Fourier coefficients with PC1_EF_ explaining 53.69% and PC2_EF_ explaining 10.17% of the variance (Figure 3). For Carcharhinidae and Sphyrnidae PC1_EF_ and PC2_EF_ scores, Carcharhinus, Sphyrna and Eusphyra, and Prionace were the genera that overlapped least in morphological (or PCA) space (Figure 3). Many of the single taxa genera in our Carcharinids and Sphyrnids species pool, such as *Galeocerdo cuvier* (Tiger Shark), *Isogomphodon oxyrhynchus* (Daggernose Shark), *Lamiopsis temminkcii* (Broadfin Shark), *Loxodon macrorhinus* (Sliteye Shark), *Nasolamia velox* (Whitenose Shark), and *Triaenodon obesus* (Whitetip Reef Shark), had positive PC2_EF_ scores for most of their pictographs and overlapped with a subsection of the Carcharhinus morphospace (Figure 3). Eccentricity and convexity interacted across PC1_EF_ and PC2_EF_, with more eccentric pictographs having positive PC1_EF_ and negative PC2_EF_ scores (Figure S6c) while more convex pictographs had negative PC1_EF_ and positive PC2_EF_ scores (Figure S6d).

### 3.2 Pictograph validation

We collated standardized length measurements from the literature for all 15 Lamniformes species from 21 sources collected across 125 specimens (Data S1). These measurements were from rostrum tip to the first dorsal fin origin, to the pelvic fin origin, to the second dorsal fin origin, to the anal fin origin, and to the upper caudal fin origin as well as the total length of the first dorsal, second dorsal, and anal fins (Figure 1). We were able perform the same measurements on 126 of the 133 (95%) Lamniformes pictographs. *Alopias pelagicus* (Pelagic Thresher), had the lowest specimen measurement coverage (*n* = 3, 37.5%) while five of the 15 species had all eight measurements (Figure 1). Generally, Lamnid species had positive residuals between the specimen measurements and the pictograph measurements indicating their pictographs underestimated the proportion of total length while for *Alopias superciliosus* (Bigeye Thresher), *Carcharias taurus* (Sand Tiger Shark), *Cetorhinus maximus* (Basking Shark), *Mitsukurina owstoni* (Goblin Shark), and *Pseudocarcharias kamoharai* (Crocodile Shark) the pictographs overestimated the proportion of total length relative to the specimen measurements (Figure 1). These discrepancies arise most frequently in the measurements from rostrum tip to second dorsal fin origin, to the pelvic origin, and to the anal fin origin and infrequently in the rostrum tip to first dorsal fin origin as well as in the fin length measurements (Figure 1). Nonetheless, these residuals, on average −0.31% across all species and measurements, are well within the range of proportional measurements from actual specimens, on average 1.61% across all species and measurements (Ahnelt et al., 2020; Cooper et al., 2020; Yano et al., 2007; Data S1). This validates the collated pictographs as representative of actual specimens and bolsters the use of pictographs as morphological trait metrics.

### 3.3 Pictograph assessments

#### 3.3.1 Assessing variability from habitat preference

Variation in global shape measurements was minimally affected by our metrics of species availability (Table 1), assessed as endemism and habitat association (Table S4). Pelagic species, including epi‐, meso‐, and bathypelagic classes, and benthic species had a significantly higher number of pictographs while those belonging to the nursery habitat class or those that had higher endemism had significantly lower numbers of pictographs (Table 1). This is not surprising given that most pelagic species have circumglobal distributions resulting in low endemism. For eccentricity, only species belonging meso‐ or bathypelagic habitat classes had significantly higher variation in eccentricity while species with higher endemism had significantly lower variation (Table 1). Species in the inland habitat class had significantly higher variation in Haralick's circularity (Table 1). Similar to the number of pictographs, the pelagic, epi‐, and mesopelagic species had significantly lower variability in the EF dissimilarity ratio.

**TABLE 1:** Effect sizes for the habitat classes and endemism (percent ocean's area covered by a species distribution) for the number of pictographs $\left(N_{\text{picts}}\right)$, the standard deviation of global shape measurements of eccentricity, Haralick's circularity, and convexity, as well as the Elliptical Fourier (EF) coefficients dissimilarity ratio

	Covariate	$N_{\text{picts}}$	Eccentricity	Haralick's circularity	Convexity	EF dissimilarity ratio
Habitat classes	Neritic	−0.644	−0.239	−0.269	0.316	0.54
	Pelagic	1.245*	0.276	−0.025	0.023	−0.604*
	Inland	−0.676	0.086	0.436*	0.1	−0.824
	Benthic	0.883*	0.063	0.076	−0.101	−0.497
	Nursery	−0.75*	−0.009	0.142	0.028	0.365
	Epipelagic	1.245*	0.276	−0.025	0.023	−0.604*
	Mesopelagic	1.177*	0.3*	−0.007	0.112	−0.633*
	Bathypelagic	0.84*	0.418*	0.131	−0.071	−0.612
	Endemism	−2.29*	−0.601*	−0.114	−0.432	0.969

*Note*: Significant effects (*p* < .05) are denoted by asterisks.

#### 3.3.2 Assessing variability from illustrators

After controlling for source and intraspecies variability (Figure S8), the greatest difference between illustrators was in the eccentricity and Haralick's circularity of the pictographs (Table 2). For the global shape measurements, negative effect sizes for eccentricity indicate more elongatedness, positive effect sizes for Haralick's circularity indicate more compactness, and negative effect sizes for convexity indicate a smaller ratio of the pictograph's area to its convex hull's area. With the exception of Marc Dando (Compagno et al., 2005; Ebert et al., 2021), the significant eccentricity effects were all negative indicating a systematic elongation in the pictographs of the Rafael de Alcantara Brandi, Emanuela D'Antoni, and O. Lidonnici relative to the other illustrators (Table 2). Similarly, all significant circularity effects were negative except for Diane Rome Peebles' pictographs indicating that Emanuela D'Antoni, Toshijii Kamohara, and O. Lidonnici's pictographs were less compact that other illustrators (Table 2). No illustrator had significant convexity effects.

**TABLE 2:** Effect sizes for each illustrator for the global shape measurements (eccentricity, Haralick's circularity, and convexity) and the Elliptical Fourier (EF) coefficients dissimilarity ratio

Abbv.	Illustrator	Eccentricity	Haralick's circularity	Convexity	EF dissimilarity
RdAB	Rafael de Alcantara Brandi	−0.321*	−0.286	0.248	−1.37*
MD	Marc Dando	0.256*	0.182	−0.364	−1.165*
DBS	D. Bryan Stone III	0.083	0.124	0.25	−1.18*
DRP	Diane Rome Peebles	0.312	0.793*	0.518	−1.2*
TK	Toshijii Kamohara	−0.557	−0.752*	−0.703	−0.794*
LJVC	Leonard J.V. Compagno	−0.006	−0.337	−0.508	−1.495*
PL	P. Lastrico	−0.113	−0.108	0.027	−1.03*
ED	Emanuela D'Antoni	−0.28*	−0.449*	−0.248	−1.353*
OL	O. Lidonnici	−0.465*	−0.841*	0.11	−1.221*
Opic	Opic	−0.165	−0.182	0.026	−1.135*
RS	Roger Swainston	−0.069	0.162	0.375	−1.208*

*Note*: Significant effects (*p* < .05) are denoted by asterisks.

Significant illustrator effects did not necessarily translate to the significant pairwise differences (Figure S7). For instance, Diane Rome Peebles' (Castro, 2011), P. Lastrico's (Compagno, 1984a, 1984b), and Roger Swainston's (Last & Stevens, 2009) pictographs were significantly different from four, six, and four other illustrators of the 11 total for eccentricity, respectively (Figure S8). Toshijii Kamohara's pictographs (Kamohara, 1967) were the most different from all other pictographs in convexity and significantly different between him and Diane Rome Peebles' and Roger Swainston's pictographs (Castro, 2011; Last & Stevens, 2009; Figure S7). The few significant effects, limited significant pairwise differences, and small effect sizes imply that, across the species pool, illustrators tend not to be exceptionally systematically variable relative to one another. This does not mean that there is no systematic variability, as many of the illustrators had effect sizes that differed from zero.

### 3.4 Applications

#### 3.4.1 Ancestral trait reconstruction

**FIGURE 4:**
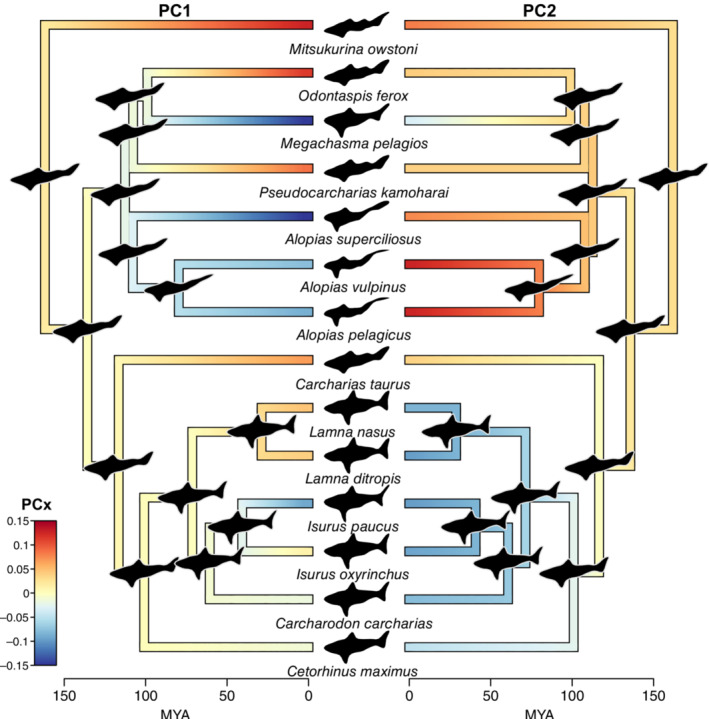
Continuous mapping on the same Lamniformes phylogeny of the PC1_EF_ (left phylogeny) and PC2_EF_ (right phylogeny) scores from the PCA on the Elliptical Fourier coefficients (negative PC_X_ scores in blue and positive PC_X_ scores in orange). The lateral profiles from the PC1 & 2 centroid for each extant species are provided in the middle while the ancestral trait reconstruction of the lateral profiles is provided at each node of the phylogeny (note the lateral profiles and phylogeny are reflected on the left and right, only the PC_EF_ mapping changes).

The reconstructions of the Lamniform ancestral morphospace progressed from the tip trait values backwards in time to the mean PC1_EF_ and PC2_EF_ scores, with the deepest node of the phylogeny being slightly positive on PC1_EF_ and PC2_EF_ (0.014, 0.021) (Figure S9). Based on morphospace back‐calculation of the PC1_EF_ and PC2_EF_ scores for the clade of Lamnidae and Cetorhinidae, the erect first dorsal fins, high aspect ratio caudal fins, large caudal fin heterocercal angle (sensu Thomson & Simanek, 1977), and reduced anal and second dorsal fins of extant species persisted until roughly 100 mya (Figure 4). Moving backwards from the tips for this clade, the first dorsal, and the ventral caudal lobe all reduced in size and were reflected in increasing PC2_EF_ scores. For the clade of Alopiidae, Pseudocarcharhidae, Megachasmidae, and Odontaspis, the less first dorsal fins, more erect second dorsal fins, smaller heterocercal angle, and larger upper caudal lobe of extant species persisted to the deepest node (~160 mya) (Figure 4). *Mitsukurina owstoni*, *Pseudocarcharias kamoharai*, and Odontaspis species with more pronounced second dorsal and anal fins had positive PC1_EF_ scores that decreased toward zero while *Megachasma pelagios* (Megamouth Shark) and Alopias species with reduced second dorsal and anal fins had negative PC1_EF_ scores that increased toward zero moving backward in time. Apart from *Megachasma pelagios*, all other species in this clade had positive PC2_EF_ scores, indicating a more elongated lateral profile that decreased moving backward in time.

#### 3.4.2 Phylogenetic generalized linear mixed model

**FIGURE 5:**
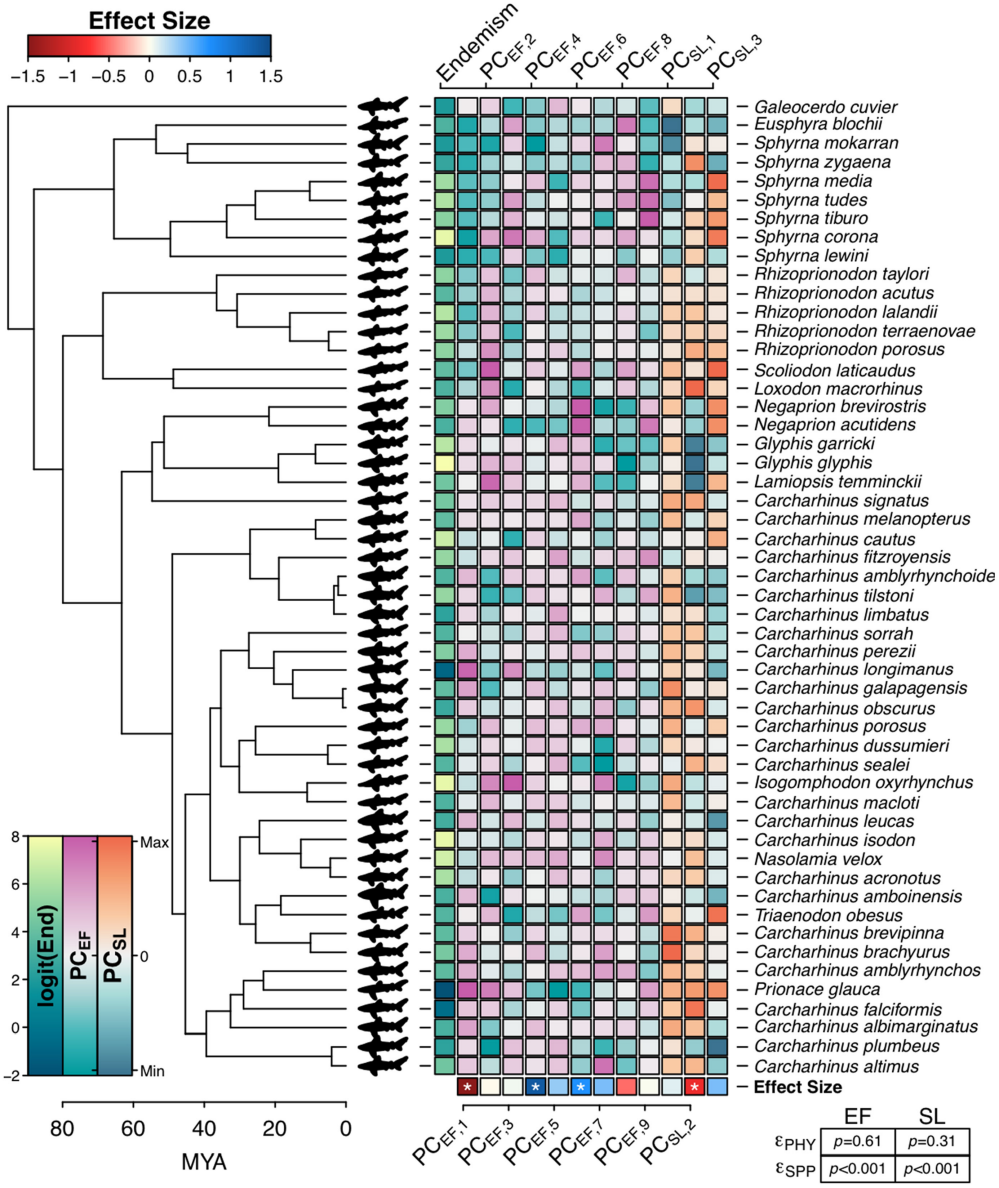
Carcharhinidae and Sphyrnidae phylogeny with each species' lateral profile, logit‐transformed endemism (logit(End)) (warmer colors indicate higher endemism), principal component scores from significant axes of the PCA on the Elliptical Fourier coefficients (PC_EF_, negative scores in teal and positive scores in pink), principal components scores from significant axes of the PCA on the standardized length measurements (PC_SL_, negative scores in blue and positive scores in orange). At the bottom of the heatmap are the effect sizes from two phylogenetic generalized linear mixed models, one using the PC_EF_ scores and the other using the PC_SL_ scores (red indicates negative values, blue indicates positive values, and asterisks indicate significant effects). In the lower right, the *p*‐values for the PGLMM unconstrained species random effect $\left(\varepsilon_{\mathrm{SPP}}\right)$ and the phylogenetically constrained species random effect $\left(\varepsilon_{\mathrm{PHY}}\right)$ is provided.

Both phylogenetic generalized mixed models of endemism, one on PC_EF_ and another on PC_SL_, had significant random effects for the unconstrained but not for the phylogenetically constrained species random effects (Figure 5). In the PC_EF_ PGLMM, the effect for PC1_EF_ was significantly negative indicating that increases in the PC_EF_ scores resulted in less endemism while the significant positive effects in PC4_EF_ and PC6_EF_ indicate that increases in these PC_EF_ scores result in more endemism (Figure 5). In species with very low endemism, such as *Prionace glauca*, the PC1_EF_ scores were strongly positive while scores for PC4_EF_ and PC6_EF_ were negative. For some species with very high endemism, such as *Isogomphodon oxyrhynchus*, *Nasolamia velox*, and *Sphyrna corona* (Scalloped Bonnethead Shark), these scores were the opposite (−PC1_EF_, +PC4_EF_, +PC6_EF_) while for others, such as the Glyphis species, PC_EF_ 1, 4, and 6 were all positive. This is consistent with expectations from the species ranges with *Prionace glauca* exhibiting a circumglobal range while Glyphis species and many of the single species Carcharhinidae genera are confined to subregions of oceanic basins or coastlines (e.g., the tropical east Pacific seaboard for *Nasolamia velox*) (Ebert et al., 2021).

The PC1_EF_ axis translates to reduced second dorsal, pelvic, and anal fins for positive values as well as a more elongated anterior trunk (before the first dorsal fin) (e.g., a *Carcharhinus longimanus*, Oceanic Whitetip Shark, lateral profile) (Figure 3). In PC4_EF_, negative scores corresponded to more acute angle between the anterior and posterior edges of the first dorsal fin, a less pronounced lower caudal lobe, and smaller caudal fin heterocercal angle (e.g., a *Sphyrna mokarran* lateral profile). Positive PC4_EF_ scores corresponded to a more obtuse angle for the first dorsal fin, a more pronounced lower caudal lobe, and a larger heterocercal angle (e.g., a *Carcharhinus brachyurus*, Bronze Whaler, lateral profile) (Figure S10). In PC6_EF_, negative scores corresponded to a reduced trunk height without pronounced second dorsal, pelvic, or anal fins (e.g., a *Carcharhinus falciformis*, Silky Shark, lateral profile) while positive scores corresponded to a stouter trunk with pronounced second dorsal, pelvic, or anal fins (e.g., a Lemon Shark lateral profile, *Negaprion brevirostris*) (Figure S10).

**FIGURE 6:**
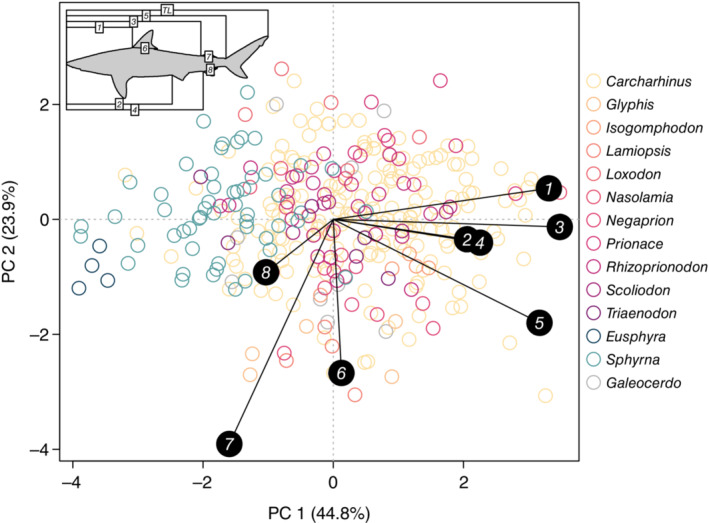
First and second principal components (PC_SL_) of the Carcharhinidae and Sphyrnidae proportional measurements (top left). The colored circles indicate the PC_SL_ scores for individual pictographs colored by genera. Solid black circle arrows indicate the loading of specific measurements (scaled to match the range of scores). The percent variance explained of the two PC_SL_ axes is labeled on the *x* and *y* axes.

We were able to perform the standardized length measurements on all 340 Carcharhinidae + Sphyrnidae pictographs. Relative to the PCA on the Elliptical Fourier coefficients, the PCA on the standardized length measurements (proportion of total length) had more overlap between all Carcharhinidae + Sphyrnidae genera with three significant PC_SL_ retained (Figure 6). The first PC_SL_ explained 44.8% while the second explained 23.9% of the total variance. Measurements from the rostrum tip to the first dorsal fin origin, to the second dorsal fin origin, and to the upper caudal fin origin strongly loaded on PC1_SL_ while total fin lengths strongly loaded on PC2_SL_ (Figure S11). In the PC_SL_ PGLMM, only PC2_SL_ had a significant negative effect on endemism (Figure 5). This corresponded to species with longer total fin length measurements with negative PC2_SL_ scores (Figure S11) generally having higher endemism, such as the Glyphis species, *Isogomphodon oxyrhynchus*, *Carcharhinus macloti* (Hardnose Shark) (Figure 5). This was not always the case as with the positive PC2_SL_ scores for *Sphyrna corona*, *Carcharhinus isodon* (Finetooth Shark), and *Nasolamia velox* despite high endemism. The PC_EF_ PGLMM had a lower log‐likelihood and better fit (*LL* = 68.5, *R* ^2^ = 0.65) than the PC_SL_ PGLMM (*LL* = 96.7, *R* ^2^ = 0.14) and, correspondingly, was significantly better when assessed using a log‐likelihood ratio test (*p* < .001). The PC_SL_ PGLMM tended to overpredict the endemism of sharks with low endemism and underpredict the endemism of sharks with very high endemism (Figure S11).

## 4 DISCUSSION

We demonstrated methods to capture, process using geometric morphometrics and reconstructive techniques, and quantitatively analyze pictographs for use as morphological traits. Global shape measurements, such as eccentricity, circularity, and convexity, provided information on broad changes in a species' morphospace while Fourier transformation and multivariate decomposition provided information on fine‐scale changes. Decomposing millions of outline coordinates to hundreds of global shape measurements or tens of thousands EFA coefficients and thousands of retained PC scores enabled a quantification of typically qualitative, discrete traits (Baken et al., 2021; Irschick et al., 2017). This reduced the subjectivity of qualitative categories, facilitated the discernment of the principal features that change across shapes, and enhanced the trait information for downstream analyses.

### 4.1 Sources of variation

Species habitat preference, as measured by habitat association and endemism, minimally impacted the global shape measurement variability but had strong effects on the number of pictographs and the variability in the Elliptical Fourier reconstruction (Table 1). The majority of habitat classes had significant effects with pelagic and benthic habitat associations increasing the number of pictographs and decreasing EF reconstruction variability while the opposite occurred with nursery habitat association. Neither the neritic nor the inland habitat associations had significant effects, which we attribute to where rare species occur across the habitat classes. While deepwater sharks are broadly more difficult to obtain access to, most of the rare species in our species pool are nearshore Carcharhinids or Sphyrnids (Figure S8). For instance, four of the 11 elasmobranch species describe in Borneo by Last et al. (2010) were Carcharhinids inhabiting nearshore waters or estuaries while the “river shark” Glyphis genera has been revised several times in the last two decades due to new descriptions (Compagno et al., 2005; Ebert et al., 2021; Last et al., 2010). This results in well‐represented species forming the bulk of a habitat classes' distribution of global shape measurement variability and rare, underrepresented species forming a heavy tail.

Across species, there was enough variability in the pictographs that few illustrators exhibited signs of systematic variability (Table 2). One way this could arise is from correlations between a species' features or identity and an illustrator's representation, such as an illustrator drawing longer requiem sharks but less convex batoid rays. This would result in multimodality in a given illustrator's global shape measurements across species and likely mimic the results we observed (Table 2; Figure S7). Expanding our species pool to include other elasmobranchs might result in this phenomenon and, depending on the morphological variability within a species pool, could be a consideration for the broader application of pictographs as morphological traits. However, our limited species pool with roughly similar lateral profiles likely limits the opportunities where an illustrator's representation would strongly correlate with a species' features. Thus, it is highly likely the limited differences we observed between illustrators are a result of minor differences in the species' scientific illustration. The PCA morphospace resulting from the Elliptical Fourier coefficients visually confirms this with relatively tight clustering for single taxa genera in the Lamniformes (Figure 2) and the Carcharhinidae + Sphyrnidae PCAs (Figure 3). Nonetheless, multiple sources proved useful in generating a central tendency in a species' representation.

After working intensely with pictographs, we feel that some of the pictograph variability is inherent in this type of data and cannot be corrected. Gathering many sources of pictographs was not necessary to achieve at least one pictograph per species (Tables S2 and S3) but this broad data collection facilitated the identification of outlier pictographs, such as the Toshijii Kamohara's drawings in Fishes of Japan in Color (Kamohara, 1967; Figure S7). While more pictographs can increase intraspecific variation, we found that most pictographs for a given species were very similar across global shape measurements (Figure S8) and had similar EF dissimilarity ratios. More sources can also help overcome the variation that results from species rarity or endemism. However, the inclusion of more pictographs may necessitate the need to filter and remove pictographs that disproportionately inflate intra‐ and interspecies variability. We purposely only filtered completely redundant sources to facilitate assessing the diversity of species' pictographic representations. This decision may marginally bias our application results by allowing some replicates (i.e., the same pictograph reprinted in other sources) and “spurious” pictographs into the total set.

### 4.2 Utility of pictographs

From our experience with two shark lineages, we feel that pictographs are worth the potential variability and challenges. At the simplest, pictographs were far easier to collate and collect from compendium sources than finding standardized length measurements. Even for frequently caught species, like *Alopias pelagicus*, we only were able to find three of the eight measurements while we collated seven illustrations. This was not unique to this species either with 10 of the 15 Lamniformes species missing at least one of the eight measurements. That is not to say these measurements are not available somewhere but, for most macroecological or phylogenetic analyses, the difficulty in rapidly collating these measurements is a hindrance to their use. Perhaps, compendium databases such as FishBase will add such measurements down the line as trait‐based analyses proliferate.

In our comparison between principal components derived from Elliptical Fourier reconstruction and those derived from standardized length measurements, the former had a better fit to the endemism data and produced more reasonable predictions from just the significant effects (Figure S11). This is more than likely from the multicollinearity in the length measurements with measurements from rostrum tip (measures 1–5) having mostly strong positive correlations and measurements of total fin length (measures 6–8) having moderate to strong positive correlations (Figure S12). Multicollinearity is consistent with other studies (Irschick et al., 2017) and results in the retention of fewer significant PCs and less significant effects in the standardized length model (Figure 5). This contrasts with the PCA of the Elliptical Fourier coefficients which had nine significant PCs. The greater performance of the shape reconstruction model (PC_EF_) over the standardized length model (PC_SL_) is further reinforced by examining the data. While smaller shark species (maximum length < 150 cm) tend to have high endemism (e.g., the Sharpnose and Spadenose Sharks; *Rhizopriondon spp*. and *Scoliodon laticaudus*, respectively), the endemism of larger sharks (maximum length > 200 cm) spanned from very low (e.g., Blue Shark) to high (e.g., River Sharks; *Glyphis spp*.). The lateral profile trait suite captured this variation in large shark endemism through the elongation and narrowing of fins and the trunk in low endemism species while high endemism species had stockier bodies with wider fins, especially in the second dorsal and pelvic fins. As a morphological trait, lateral profile pictographs provided a much richer information source to ecological and evolutionary analyses than more traditional length measurements.

By back‐calculating the pictograph from a given PC_EF_ score, we developed a method to relate the multivariate morphospace back to a pictographic representation. This extension facilitates a broader use of geometric reconstructive techniques in community phylogenetics, comparative phylogenetics, and functional trait analyses. In our ancestral trait reconstruction example, we were able to track the change in PC1_EF_ and PC2_EF_ scores backwards in time and relate these changes specifically to the lateral profile. To generate a more realistic reconstructed lateral profile, we could have extended this approach to all seven significant PCs for the Lamniformes PCA. Nonetheless, by applying this approach, we were able to visualize that the profile commonly associated with Lamniforms and exhibited by Great White Sharks is a relatively new trait with respect to the clade's divergence from other shark lineages. Morphological measurements, with their inherent multicollinearity and information loss relative to a full lateral profile, are unlikely to easily garner such insights.

Like all traits, the lateral profile trait set we assembled from the pictograph collection and processing is not a catch‐all for the evolutionary history or trait diversity of our species pool (Palci & Lee, 2019). Instead, it should be used alongside other traits that capture different aspects of morphological or functional diversity (Moen, 2019). There are instances where this is readily apparent. For example, our morphospace of Carcharhinid and Sphyrnid lateral profiles shows that there is strong conservation of the requiem shark body shape with minimum changes (e.g., elongating or widening of fins, tilting of the caudal fin, elongation of the rostrum, and deepening of the trunk) (Irschick et al., 2017; Sternes & Shimada, 2020). However, this fails to capture the massive anatomical differences in the heads of Sphrynids (e.g., hammerheads) and Carcharhinids. An additional challenge is the considerable number of degrees of freedom the lateral profile trait set, comprised of the significant PC scores, consumes in other analyses. For example, nine PCs were retained from the Carcharhinid and Sphyrnid Elliptical Fourier PCA which resulted in nine effects in the PGLMM. Smaller species pools or analyses with many traits could easily run into issues where there are too many traits for a given analysis. There are several solutions to this, but the easiest is to willingly accept a higher threshold for significance that, in turn, reduces the number of PCs retained. This is not a feature inherent to the use of pictographs and one faced broadly across principal components regressions (Lang & Zou, 2020).

### 4.3 Collation and processing challenges

We encountered a few challenges in the collation and processing of the pictographs that are not fully captured in our enumeration and quantification methods. One challenge was perspective. If we were to extend our shark examples to a broader set of elasmobranch species, we are likely to run into issues where some species (e.g., Squantinids and Batoidea) only have dorsal perspectives of body shape (Siders et al., 2022). Conversely, we generally lacked dorsal perspectives in our pictograph sources. Another challenge was jaw representation. Several illustrators tended to illustrate one or two species with open jaws rather than closed jaws (e.g., *Megachasma pelagios*) which alter the lateral profile, predominantly in lowering the eccentricity measurement. The depiction of different life stages, such as drawings of pregnant females, was a relatively minor challenge resulting in a few pictographs severely deviating from the mean for the species. Additionally, we avoided some ancillary drawings in the capture phase such as Diane Rome Peebles' depiction of ecomorphs or juvenile life stages in Castro (2011). In the processing phase, we trialed several alignment methods before settling on landmarking. The landmarking process requires more work to implement pictographs as morphological traits as well as introduces a subjective choice and potential bias.

More broadly, we encountered considerable amounts of republishing across the sources we gathered. Even in the pictograph set we used, we had duplicate pictographs. We could have removed duplicates in the collection phase, but a more prudent measure would be to use the global shape measurements or the EF dissimilarity ratio within a species to identify and prune duplicates. These could also be identified as pictographs occupying the exact same location in the trait (PCA) morphospace. Another challenge we encountered was identifying the illustrator in compendium‐style sources. For example, in Compagno (1984b), there were four illustrators (plus the author himself) listed, and in Compagno (2002), there were eight illustrators listed. However, in our pictographs from these sources, we only identified four and three unique signatures, respectively, with many pictographs remaining unsigned, which forced us to assume the unsigned were from the main illustrator.

## 5 CONCLUSION

Pictographs are an overlooked but common source of scientific information. By utilizing geometric morphometric and reconstructive methods to process and analyze pictographs, we demonstrate their utility and application to a wide variety of trait‐based ecological, phylogenetic, and physiological analyses. In marine species, particularly megafauna, pictographs from scientific illustration may be the most feasible approach to obtain morphological traits as it is often difficult to obtain, store, or scan specimens which inhibits the applicability of standardized measurements. While reducing three‐dimensional objects to two‐dimensional pictographs represents an inherent loss in information, it is a vast improvement to reducing these same objects to a simple measurements. Additionally, we found that other pictograph perspectives are common throughout the literature and, generally, focus on highly specialized/different traits (e.g., teeth, jaws, etc.) though two recent studies have used pictographs in sharks (Sternes et al., 2022; Sternes & Shimada, 2020). These resources represent a readily available source of traits to capture multiple facets of morphology and are an area we intend to explore further with our shark species pool. Lastly, we found pictographs to be an area of many museum collections that is likely underutilized and feel that digitizing these archival materials can reveal the evolution of scientific thought about a species representation.

## AUTHOR CONTRIBUTIONS

**Zachary Siders:** Conceptualization (lead); data curation (equal); formal analysis (lead); funding acquisition (equal); methodology (lead); project administration (lead); visualization (lead); writing – original draft (equal); writing – review and editing (equal). **Fabio Caltabellotta:** Data curation (equal); funding acquisition (equal); writing – original draft (equal); writing – review and editing (equal). **Katherine Loesser:** Data curation (equal); writing – original draft (equal); writing – review and editing (equal). **Lauren Trotta:** Funding acquisition (equal); methodology (supporting); writing – original draft (equal); writing – review and editing (equal). **Benjamin Baiser:** Funding acquisition (equal); writing – original draft (equal); writing – review and editing (equal).

## CONFLICT OF INTEREST

The authors have no conflicts of interest to declare.

## Supporting information


Appendix S1.

Data S1.

## Data Availability

The processed pictographs, covariates, phylogenies, and scripts are reposited in an Open Science Framework repository at https://osf.io/mzv8y/.
