# Supplementary material for: Using pictographs as traits to explore morphological diversity in sharks
Source: Ecol Evol. 2023 Jan 24;13(1):e9761. doi: 10.1002/ece3.9761 (PMC9873591; doi:10.1002/ece3.9761)
Supplement: Supplementary file 1 — Appendix S1. [file ECE3-13-e9761-s001.docx]

# supplemental Information

## Supplemental Table 1 – Habitat Classification Scheme

Table indicating the translation from the IUCN Habitat Type to the habitat classes used in the study.

| Habitat Class | IUCN Habitat Type |
| --- | --- |
| Neritic | Marine Neritic – …. |
| Pelagic | Marine Oceanic – … |
| Inland | Wetlands (inland) - Permanent Freshwater Lakes (over 8ha)  Wetlands (inland) - Permanent Rivers/Streams/Creeks (includes waterfalls)  Marine Coastal/Supratidal - Coastal Brackish/Saline Lagoons/Marine Lakes |
| Benthic | Marine Deep Benthic - Continental Slope/Bathyl Zone (200-4,000m)  Marine Deep Benthic - Seamount |
| Nursery | Marine Intertidal - Mangrove Submerged Roots  Marine Intertidal - Sandy Shoreline and/or Beaches, Sand Bars, Spits, Etc,  Marine Intertidal - Shingle and/or Pebble Shoreline and/or Beaches  Marine Neritic – Estuaries  Marine Neritic - Coral Reef  Marine Neritic - Seagrass (Submerged) |
| Epipelagic | Marine Oceanic - Epipelagic (0-200m) |
| Mesopelagic | Marine Oceanic - Mesopelagic (200-1000m) |
| Bathypelagic | Marine Oceanic - Bathypelagic (1000-4000m) |

## Supplemental Table 2 – Pictograph Sources

Pictograph species and sources with the illustrator initials below and the total pictographs per species. A line indicates the break between Lamniformes (above) and Carcharinids and Sphyrnids (below).

| Species | FJC (1967) | RdMA (1981) | SoNA (1983) | FAO.4.2 (1983) | SRA (1994) | FAO.1.2 (2002) | TDB (2002) | SoW (2005) | SoNA (2011) | SRNA (2013) | FAO.Reg (2015,16) | SoW (2021) | Total |
| --- | --- | --- | --- | --- | --- | --- | --- | --- | --- | --- | --- | --- | --- |
|  | TK | O | DBS | EA+^1^ | RS | EA+^1^ | RdAB | MD | DRP | EA+^1^ | EA+^1^ | MD | – |
| *Alopias pelagicus* | 1 | 0 | 1 | 0 | 1 | 1 | 0 | 1 | 1 | 0 | 0 | 1 | 7 |
| *Alopias superciliosus* | 0 | 1 | 1 | 0 | 1 | 1 | 1 | 1 | 1 | 1 | 1 | 1 | 10 |
| *Alopias vulpinus* | 0 | 1 | 1 | 0 | 1 | 1 | 1 | 1 | 1 | 1 | 1 | 1 | 10 |
| *Carcharias taurus* | 0 | 1 | 1 | 0 | 0 | 1 | 1 | 1 | 1 | 1 | 1 | 1 | 9 |
| *Carcharodon carcharias* | 0 | 1 | 1 | 0 | 1 | 1 | 1 | 1 | 1 | 1 | 1 | 1 | 10 |
| *Cetorhinus maximus* | 0 | 1 | 1 | 0 | 1 | 1 | 1 | 1 | 1 | 1 | 1 | 1 | 10 |
| *Isurus oxyrinchus* | 1 | 1 | 1 | 0 | 1 | 1 | 1 | 1 | 1 | 1 | 2 | 1 | 12 |
| *Isurus paucus* | 0 | 1 | 0 | 0 | 1 | 1 | 1 | 1 | 1 | 1 | 1 | 1 | 9 |
| *Lamna ditropis* | 1 | 1 | 0 | 0 | 1 | 1 | 0 | 1 | 1 | 0 | 0 | 1 | 7 |
| *Lamna nasus* | 0 | 1 | 1 | 0 | 1 | 1 | 1 | 1 | 1 | 1 | 1 | 1 | 10 |
| *Megachasma pelagios* | 0 | 0 | 1 | 0 | 1 | 1 | 1 | 1 | 1 | 0 | 0 | 1 | 7 |
| *Mitsukurina owstoni* | 0 | 1 | 1 | 0 | 1 | 1 | 1 | 1 | 1 | 1 | 1 | 1 | 10 |
| *Odontaspis ferox* | 0 | 1 | 1 | 0 | 1 | 1 | 1 | 1 | 1 | 1 | 0 | 1 | 9 |
| *Odontaspis noronhai* | 0 | 1 | 0 | 0 | 0 | 1 | 1 | 1 | 1 | 0 | 0 | 1 | 6 |
| *Pseudocarcharias kamoharai* | 0 | 1 | 1 | 0 | 0 | 1 | 1 | 1 | 1 | 0 | 0 | 1 | 7 |
| *Carcharhinus acronotus* | 0 | 1 | 0 | 1 | 1 | 0 | 1 | 1 | 1 | 1 | 1 | 1 | 9 |
| *Carcharhinus albimarginatus* | 0 | 0 | 1 | 1 | 0 | 0 | 0 | 1 | 1 | 0 | 0 | 1 | 5 |
| *Carcharhinus altimus* | 0 | 1 | 1 | 1 | 1 | 0 | 1 | 1 | 1 | 1 | 1 | 1 | 10 |
| *Carcharhinus amblyrhynchoides* | 0 | 0 | 1 | 1 | 0 | 0 | 0 | 1 | 0 | 0 | 0 | 1 | 4 |
| *Carcharhinus amblyrhynchos* | 0 | 0 | 1 | 1 | 0 | 0 | 0 | 1 | 0 | 0 | 0 | 1 | 4 |
| *Carcharhinus amboinensis* | 0 | 1 | 1 | 1 | 0 | 0 | 0 | 1 | 0 | 0 | 0 | 1 | 5 |
| *Carcharhinus brachyurus* | 0 | 1 | 1 | 1 | 1 | 0 | 1 | 1 | 1 | 0 | 0 | 1 | 8 |
| *Carcharhinus brevipinna* | 0 | 1 | 1 | 1 | 1 | 0 | 1 | 1 | 1 | 1 | 1 | 1 | 10 |
| *Carcharhinus cautus* | 0 | 0 | 1 | 1 | 0 | 0 | 0 | 1 | 0 | 0 | 0 | 1 | 4 |
| *Carcharhinus dussumieri* | 0 | 0 | 1 | 1 | 0 | 0 | 0 | 1 | 0 | 0 | 0 | 1 | 4 |
| *Carcharhinus falciformis* | 0 | 1 | 1 | 1 | 1 | 0 | 1 | 1 | 1 | 1 | 1 | 1 | 10 |
| *Carcharhinus fitzroyensis* | 0 | 0 | 1 | 1 | 0 | 0 | 0 | 1 | 0 | 0 | 0 | 1 | 4 |
| *Carcharhinus galapagensis* | 0 | 1 | 1 | 1 | 1 | 0 | 1 | 1 | 1 | 0 | 0 | 1 | 8 |
| *Carcharhinus isodon* | 0 | 1 | 0 | 1 | 1 | 0 | 1 | 1 | 1 | 1 | 0 | 1 | 8 |
| *Carcharhinus leucas* | 0 | 1 | 1 | 1 | 1 | 0 | 1 | 1 | 1 | 1 | 1 | 1 | 10 |
| *Carcharhinus limbatus* | 0 | 1 | 1 | 1 | 1 | 0 | 1 | 1 | 1 | 1 | 1 | 1 | 10 |
| *Carcharhinus longimanus* | 0 | 0 | 1 | 1 | 1 | 0 | 1 | 1 | 1 | 1 | 1 | 1 | 9 |
| *Carcharhinus macloti* | 0 | 1 | 1 | 1 | 0 | 0 | 0 | 1 | 0 | 0 | 0 | 1 | 5 |
| *Carcharhinus melanopterus* | 0 | 1 | 1 | 1 | 0 | 0 | 0 | 1 | 0 | 0 | 0 | 1 | 5 |
| *Carcharhinus obscurus* | 0 | 1 | 1 | 1 | 1 | 0 | 1 | 1 | 1 | 1 | 1 | 1 | 10 |
| *Carcharhinus perezii* | 0 | 1 | 0 | 1 | 1 | 0 | 1 | 1 | 1 | 0 | 1 | 1 | 8 |
| *Carcharhinus plumbeus* | 0 | 1 | 1 | 1 | 1 | 0 | 1 | 1 | 1 | 1 | 1 | 1 | 10 |
| *Carcharhinus porosus* | 0 | 0 | 0 | 1 | 1 | 0 | 1 | 1 | 1 | 0 | 0 | 1 | 6 |
| *Carcharhinus sealei* | 0 | 0 | 0 | 1 | 0 | 0 | 0 | 1 | 0 | 0 | 0 | 1 | 3 |
| *Carcharhinus signatus* | 0 | 1 | 0 | 1 | 1 | 0 | 1 | 1 | 1 | 1 | 1 | 1 | 9 |
| *Carcharhinus sorrah* | 0 | 0 | 1 | 1 | 0 | 0 | 0 | 1 | 0 | 0 | 0 | 1 | 4 |
| *Carcharhinus tilstoni* | 0 | 0 | 1 | 0 | 0 | 0 | 0 | 1 | 0 | 0 | 0 | 1 | 3 |
| *Eusphyra blochii* | 0 | 1 | 0 | 1 | 0 | 0 | 0 | 1 | 0 | 0 | 0 | 1 | 4 |
| *Galeocerdo cuvier* | 0 | 0 | 1 | 1 | 1 | 0 | 1 | 1 | 1 | 1 | 1 | 1 | 9 |
| *Glyphis garricki* | 0 | 0 | 0 | 0 | 0 | 0 | 0 | 1 | 0 | 0 | 0 | 1 | 2 |
| *Glyphis glyphis* | 0 | 0 | 1 | 1 | 0 | 0 | 0 | 1 | 0 | 0 | 0 | 1 | 4 |
| *Isogomphodon oxyrhynchus* | 0 | 1 | 0 | 1 | 0 | 0 | 1 | 1 | 1 | 0 | 0 | 1 | 6 |
| *Lamiopsis temminckii* | 0 | 1 | 0 | 1 | 0 | 0 | 0 | 1 | 0 | 0 | 0 | 1 | 4 |
| *Loxodon macrorhinus* | 0 | 1 | 1 | 1 | 0 | 0 | 0 | 1 | 0 | 0 | 0 | 1 | 5 |
| *Nasolamia velox* | 0 | 0 | 0 | 1 | 1 | 0 | 0 | 1 | 1 | 0 | 0 | 1 | 5 |
| *Negaprion acutidens* | 0 | 0 | 1 | 1 | 0 | 0 | 0 | 1 | 0 | 0 | 0 | 1 | 4 |
| *Negaprion brevirostris* | 0 | 0 | 0 | 1 | 1 | 0 | 1 | 1 | 1 | 1 | 0 | 1 | 7 |
| *Prionace glauca* | 1 | 1 | 1 | 1 | 1 | 0 | 1 | 1 | 1 | 1 | 1 | 1 | 11 |
| *Rhizoprionodon acutus* | 0 | 1 | 1 | 1 | 0 | 0 | 0 | 1 | 0 | 0 | 0 | 1 | 5 |
| *Rhizoprionodon lalandii* | 0 | 1 | 0 | 1 | 0 | 0 | 1 | 1 | 0 | 0 | 1 | 1 | 6 |
| *Rhizoprionodon porosus* | 0 | 1 | 0 | 1 | 1 | 0 | 1 | 1 | 0 | 0 | 1 | 1 | 7 |
| *Rhizoprionodon taylori* | 0 | 1 | 1 | 1 | 0 | 0 | 0 | 1 | 0 | 0 | 0 | 1 | 5 |
| *Rhizoprionodon terraenovae* | 0 | 1 | 0 | 1 | 1 | 0 | 0 | 1 | 1 | 1 | 1 | 1 | 8 |
| *Scoliodon laticaudus* | 0 | 1 | 0 | 1 | 0 | 0 | 0 | 1 | 0 | 0 | 0 | 1 | 4 |
| *Sphyrna corona* | 0 | 1 | 0 | 1 | 1 | 0 | 0 | 1 | 0 | 0 | 0 | 1 | 5 |
| *Sphyrna lewini* | 0 | 1 | 0 | 1 | 1 | 0 | 1 | 1 | 1 | 1 | 1 | 1 | 9 |
| *Sphyrna media* | 0 | 1 | 0 | 1 | 1 | 0 | 1 | 1 | 0 | 0 | 1 | 1 | 7 |
| *Sphyrna mokarran* | 0 | 1 | 0 | 1 | 1 | 0 | 1 | 1 | 0 | 1 | 1 | 1 | 8 |
| *Sphyrna tiburo* | 0 | 1 | 0 | 1 | 1 | 0 | 1 | 1 | 0 | 1 | 1 | 1 | 8 |
| *Sphyrna tudes* | 0 | 1 | 0 | 1 | 1 | 0 | 1 | 1 | 0 | 0 | 1 | 1 | 7 |
| *Sphyrna zygaena* | 0 | 1 | 0 | 1 | 1 | 0 | 1 | 1 | 1 | 1 | 1 | 1 | 9 |
| *Triaenodon obesus* | 0 | 1 | 1 | 1 | 0 | 0 | 0 | 1 | 1 | 0 | 0 | 1 | 6 |

^1^Emanuela D’ Antoni illustrated the majority of pictographs with a subset contributed by Paolo Lastrico, O. Lidonnici, and Leonard J.V. Compagno.

## Supplemental Table 3 – Pictographs by source and illustrator

Pictograph sources and the number of pictographs per illustrator in the source.

|  | RdAB | MD | DBS III | DRP | TK | LJVC | PL | ED | OL | Opic | RS |
| --- | --- | --- | --- | --- | --- | --- | --- | --- | --- | --- | --- |
| FJC (1967) | 0 | 0 | 0 | 0 | 4 | 0 | 0 | 0 | 0 | 0 | 0 |
| RdMA (1981) | 0 | 0 | 0 | 0 | 0 | 0 | 0 | 0 | 0 | 48 | 0 |
| SoNA (1983) | 0 | 0 | 40 | 0 | 0 | 0 | 0 | 0 | 0 | 0 | 0 |
| FAO 4.2 (1983) | 0 | 0 | 0 | 0 | 0 | 1 | 13 | 30 | 6 | 0 | 0 |
| SRA (1994) | 0 | 0 | 0 | 0 | 0 | 0 | 0 | 0 | 0 | 0 | 41 |
| FAO 1.2 (2002) | 0 | 0 | 0 | 0 | 0 | 2 | 2 | 11 | 0 | 0 | 0 |
| TDB (2002) | 40 | 0 | 0 | 0 | 0 | 0 | 0 | 0 | 0 | 0 | 0 |
| SoW (2005) | 0 | 67 | 0 | 0 | 0 | 0 | 0 | 0 | 0 | 0 | 0 |
| SoNA (2011) | 0 | 0 | 0 | 40 | 0 | 0 | 0 | 0 | 0 | 0 | 0 |
| SRNA (2013) | 0 | 0 | 0 | 0 | 0 | 2 | 3 | 23 | 1 | 0 | 0 |
| FAO Reg. (2015, 16) | 0 | 0 | 0 | 0 | 0 | 0 | 0 | 32 | 0 | 0 | 0 |
| SoW (2021) | 0 | 67 | 0 | 0 | 0 | 0 | 0 | 0 | 0 | 0 | 0 |

## Supplemental Table 4 – Assigned Habitat Memberships

Membership of each species to habitat classes derived from the IUCN habitat preferences (Supplemental Table 1). A line indicates the break between Lamniformes (above) and Carcharinids and Sphyrnids (below).

| Species | Neritic | Pelagic | Inland | Benthic | Nursery | Epipelagic | Mesopelagic | Bathypelagic |
| --- | --- | --- | --- | --- | --- | --- | --- | --- |
| *Alopias pelagicus* | 1 | 1 | 0 | 0 | 0 | 1 | 1 | 0 |
| *Alopias superciliosus* | 1 | 1 | 0 | 0 | 0 | 1 | 1 | 0 |
| *Alopias vulpinus* | 1 | 1 | 0 | 0 | 0 | 1 | 1 | 0 |
| *Carcharias taurus* | 1 | 0 | 0 | 1 | 1 | 0 | 0 | 0 |
| *Carcharodon carcharias* | 1 | 1 | 0 | 0 | 1 | 1 | 1 | 1 |
| *Cetorhinus maximus* | 1 | 1 | 0 | 0 | 0 | 1 | 1 | 1 |
| *Isurus oxyrinchus* | 0 | 1 | 0 | 0 | 0 | 1 | 1 | 0 |
| *Isurus paucus* | 0 | 1 | 0 | 0 | 0 | 1 | 1 | 1 |
| *Lamna ditropis* | 1 | 1 | 0 | 0 | 0 | 1 | 1 | 1 |
| *Lamna nasus* | 1 | 1 | 0 | 0 | 0 | 1 | 1 | 1 |
| *Megachasma pelagios* | 0 | 1 | 0 | 0 | 0 | 1 | 1 | 1 |
| *Mitsukurina owstoni* | 0 | 1 | 0 | 1 | 0 | 1 | 1 | 0 |
| *Odontaspis ferox* | 1 | 0 | 0 | 1 | 0 | 0 | 0 | 0 |
| *Odontaspis noronhai* | 0 | 1 | 0 | 0 | 0 | 1 | 1 | 0 |
| *Pseudocarcharias kamoharai* | 0 | 1 | 0 | 0 | 0 | 1 | 1 | 0 |
| *Carcharhinus acronotus* | 1 | 0 | 0 | 0 | 1 | 0 | 0 | 0 |
| *Carcharhinus albimarginatus* | 1 | 0 | 0 | 1 | 1 | 0 | 0 | 0 |
| *Carcharhinus altimus* | 1 | 1 | 0 | 1 | 0 | 1 | 0 | 0 |
| *Carcharhinus amblyrhynchoides* | 1 | 0 | 0 | 0 | 1 | 0 | 0 | 0 |
| *Carcharhinus amblyrhynchos* | 1 | 0 | 0 | 0 | 1 | 0 | 0 | 0 |
| *Carcharhinus amboinensis* | 1 | 0 | 0 | 0 | 1 | 0 | 0 | 0 |
| *Carcharhinus brachyurus* | 1 | 1 | 0 | 0 | 1 | 1 | 0 | 0 |
| *Carcharhinus brevipinna* | 1 | 0 | 0 | 0 | 0 | 0 | 0 | 0 |
| *Carcharhinus cautus* | 1 | 0 | 0 | 0 | 1 | 0 | 0 | 0 |
| *Carcharhinus dussumieri* | 1 | 0 | 0 | 0 | 0 | 0 | 0 | 0 |
| *Carcharhinus falciformis* | 1 | 1 | 0 | 1 | 0 | 1 | 1 | 0 |
| *Carcharhinus fitzroyensis* | 1 | 0 | 0 | 0 | 1 | 0 | 0 | 0 |
| *Carcharhinus galapagensis* | 1 | 1 | 0 | 0 | 1 | 1 | 1 | 0 |
| *Carcharhinus isodon* | 1 | 0 | 0 | 0 | 1 | 0 | 0 | 0 |
| *Carcharhinus leucas* | 1 | 0 | 1 | 0 | 1 | 0 | 0 | 0 |
| *Carcharhinus limbatus* | 1 | 1 | 0 | 0 | 1 | 1 | 0 | 0 |
| *Carcharhinus longimanus* | 1 | 1 | 0 | 0 | 1 | 1 | 1 | 1 |
| *Carcharhinus macloti* | 1 | 0 | 0 | 0 | 0 | 0 | 0 | 0 |
| *Carcharhinus melanopterus* | 1 | 0 | 0 | 0 | 1 | 0 | 0 | 0 |
| *Carcharhinus obscurus* | 1 | 1 | 0 | 0 | 0 | 1 | 1 | 0 |
| *Carcharhinus perezii* | 1 | 1 | 0 | 0 | 1 | 1 | 0 | 0 |
| *Carcharhinus plumbeus* | 1 | 0 | 0 | 1 | 1 | 0 | 0 | 0 |
| *Carcharhinus porosus* | 1 | 0 | 1 | 0 | 1 | 0 | 0 | 0 |
| *Carcharhinus sealei* | 1 | 0 | 0 | 0 | 0 | 0 | 0 | 0 |
| *Carcharhinus signatus* | 1 | 1 | 0 | 0 | 0 | 1 | 1 | 0 |
| *Carcharhinus sorrah* | 1 | 0 | 0 | 0 | 1 | 0 | 0 | 0 |
| *Carcharhinus tilstoni* | 1 | 0 | 0 | 0 | 0 | 0 | 0 | 0 |
| *Eusphyra blochii* | 1 | 0 | 0 | 0 | 1 | 0 | 0 | 0 |
| *Galeocerdo cuvier* | 1 | 1 | 0 | 0 | 1 | 1 | 1 | 0 |
| *Glyphis garricki* | 1 | 0 | 1 | 0 | 1 | 0 | 0 | 0 |
| *Glyphis glyphis* | 1 | 0 | 1 | 0 | 1 | 0 | 0 | 0 |
| *Isogomphodon oxyrhynchus* | 1 | 0 | 0 | 0 | 1 | 0 | 0 | 0 |
| *Lamiopsis temminckii* | 1 | 0 | 0 | 0 | 1 | 0 | 0 | 0 |
| *Loxodon macrorhinus* | 1 | 0 | 0 | 0 | 1 | 0 | 0 | 0 |
| *Nasolamia velox* | 1 | 1 | 0 | 0 | 1 | 1 | 0 | 0 |
| *Negaprion acutidens* | 1 | 0 | 0 | 0 | 1 | 0 | 0 | 0 |
| *Negaprion brevirostris* | 1 | 0 | 0 | 0 | 1 | 0 | 0 | 0 |
| *Prionace glauca* | 1 | 1 | 0 | 0 | 0 | 1 | 1 | 0 |
| *Rhizoprionodon acutus* | 1 | 0 | 0 | 0 | 1 | 0 | 0 | 0 |
| *Rhizoprionodon lalandii* | 1 | 0 | 0 | 0 | 0 | 0 | 0 | 0 |
| *Rhizoprionodon porosus* | 1 | 1 | 0 | 0 | 0 | 1 | 0 | 0 |
| *Rhizoprionodon taylori* | 1 | 0 | 0 | 0 | 1 | 0 | 0 | 0 |
| *Rhizoprionodon terraenovae* | 1 | 0 | 0 | 0 | 1 | 0 | 0 | 0 |
| *Scoliodon laticaudus* | 1 | 0 | 0 | 0 | 1 | 0 | 0 | 0 |
| *Sphyrna corona* | 1 | 0 | 0 | 0 | 1 | 0 | 0 | 0 |
| *Sphyrna lewini* | 1 | 1 | 0 | 0 | 0 | 1 | 1 | 1 |
| *Sphyrna media* | 1 | 0 | 0 | 0 | 1 | 0 | 0 | 0 |
| *Sphyrna mokarran* | 1 | 1 | 0 | 0 | 0 | 1 | 1 | 0 |
| *Sphyrna tiburo* | 1 | 0 | 0 | 0 | 1 | 0 | 0 | 0 |
| *Sphyrna tudes* | 1 | 0 | 0 | 0 | 0 | 0 | 0 | 0 |
| *Sphyrna zygaena* | 1 | 1 | 0 | 0 | 0 | 1 | 0 | 0 |
| *Triaenodon obesus* | 1 | 0 | 0 | 0 | 1 | 0 | 0 | 0 |

## Supplemental Figure 1 – General Workflow


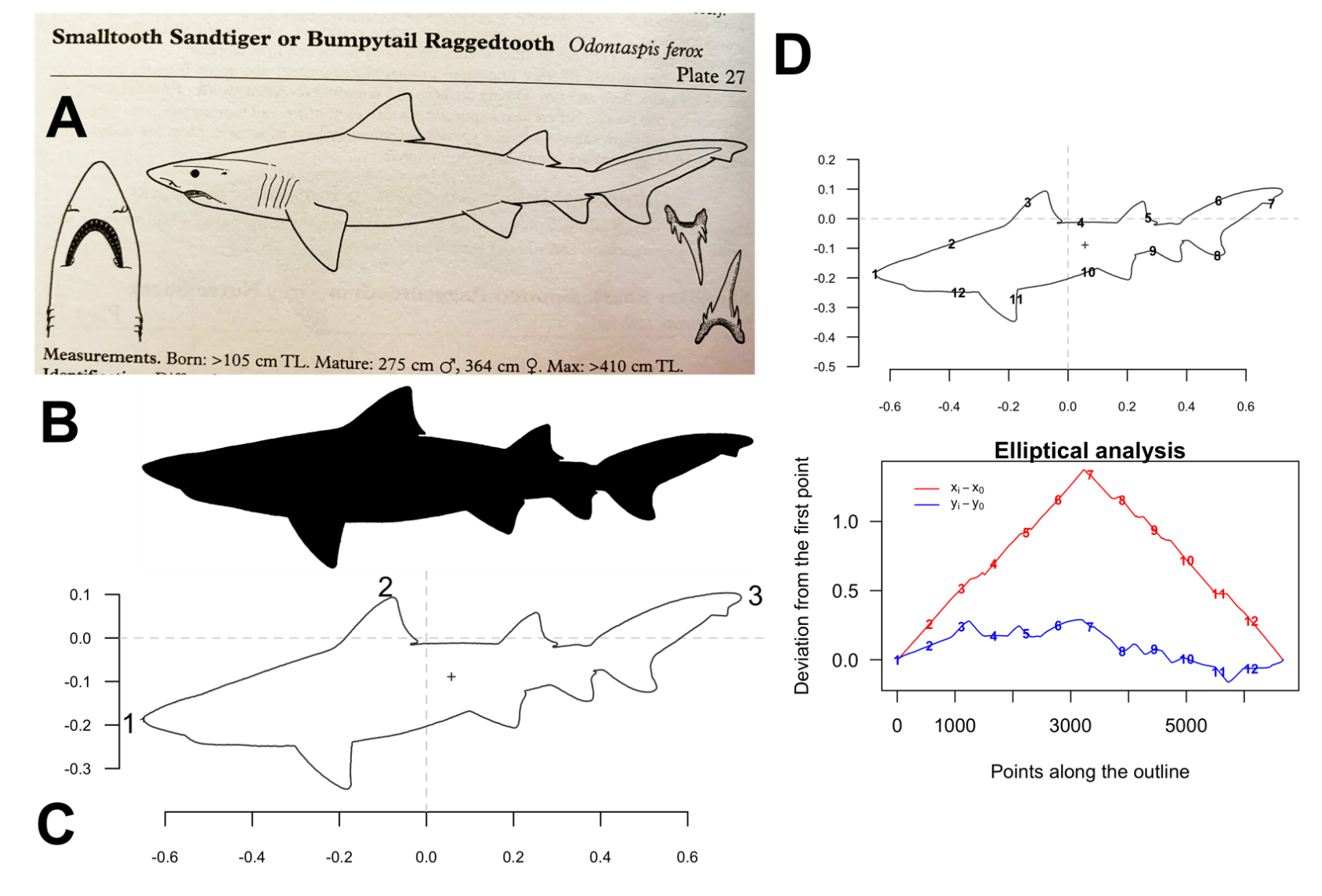


General workflow to process pictographs: (A) Pictograph of Smalltooth Sandtiger (*Odontaspis ferox*) in Sharks of the World illustrated by Marc Dando; (B) Silhouette after removing the background in Adobe Photoshop; (C) Landmarking (denoted by numbers 1-3), rescaling, and Procrustes alignment of the silhouette’s outline; (D) Elliptical Fourier Analysis conducted on outline (top) with denoted points in text (numbers 1-12), and the resulting deviation from the first point as a function of the number of points along the outline for the x and y axes (bottom).

## Supplemental Figure 2 – Frequency of pictograph sources

Number of Carcharhinidae + Sphyrnidae and Lamniformes species with a given number of pictographs (left) or a given number of illustrators (right).

## Supplemental Figure 3 – Species Discovery Curve

Species discovery curve as a function of the number of pictographs collected. The solid line is the estimated number of species for a given number of pictographs with the shaded region indicating the confidence interval. The circle indicates the total number of pictographs in our data and the dash line indicates the number of pictographs needed to obtain the estimated number of unobserved species.

## Supplemental Figure 4 – Alignment


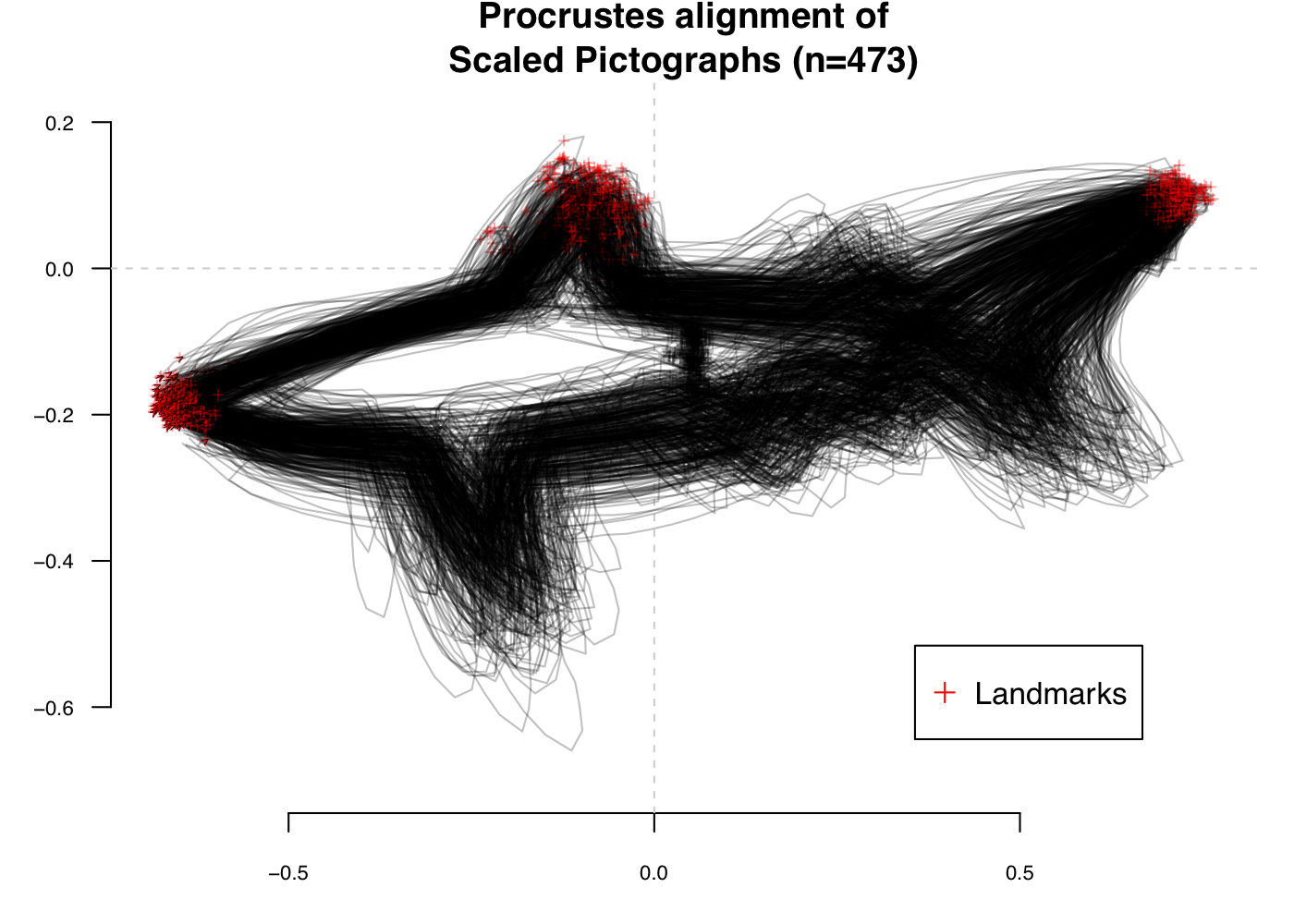


Alignment of Lamniformes and Carcharhinidae + Sphyrnidae pictographs after rescaling and Procrustes alignment.

## Supplemental Figure 5 – Elliptical Fourier Harmonics

The cumulative harmonic power as a function of the number of harmonics in the Elliptical Fourier Analysis of the Carcharhinidae + Sphyrnidae and Lamniformes species’ pictographs.

## Supplemental Figure 6 – Global Shape Measurements on PCAs

The Eigen-based eccentricity (A and C) and convexity (B and D) for the Lamniformes (A and B) and the Carcharhinidae + Sphyrnidae (C and D) PC1_EF_ and PC2_EF_ scores. Pink indicates a higher global shape measurement (i.e., more convexity or more eccentricity) while green indicates a lower global shape measurement.

## Supplemental Figure 7 – Pairwise Illustrator Comparisons

Pairwise comparisons of the expected marginal means of the illustrator effect for the global shape measurements (i.e., eccentricity, Haralick’s circularity, convexity) and the Elliptical Fourier dissimilarity. Cooler colors indicate negative pairwise differences while warmer colors indicate positive pairwise differences. Comparisons above the diagonal are the y-axis illustrator minus the x-axis illustrator and vice versa for comparisons below the diagonal, which together are the same value with opposite signs. Asterisks indicated significant pairwise differences after Bonferroni correction.

## Supplemental Figure 8 – Estimated scale from illustrator GLMMs


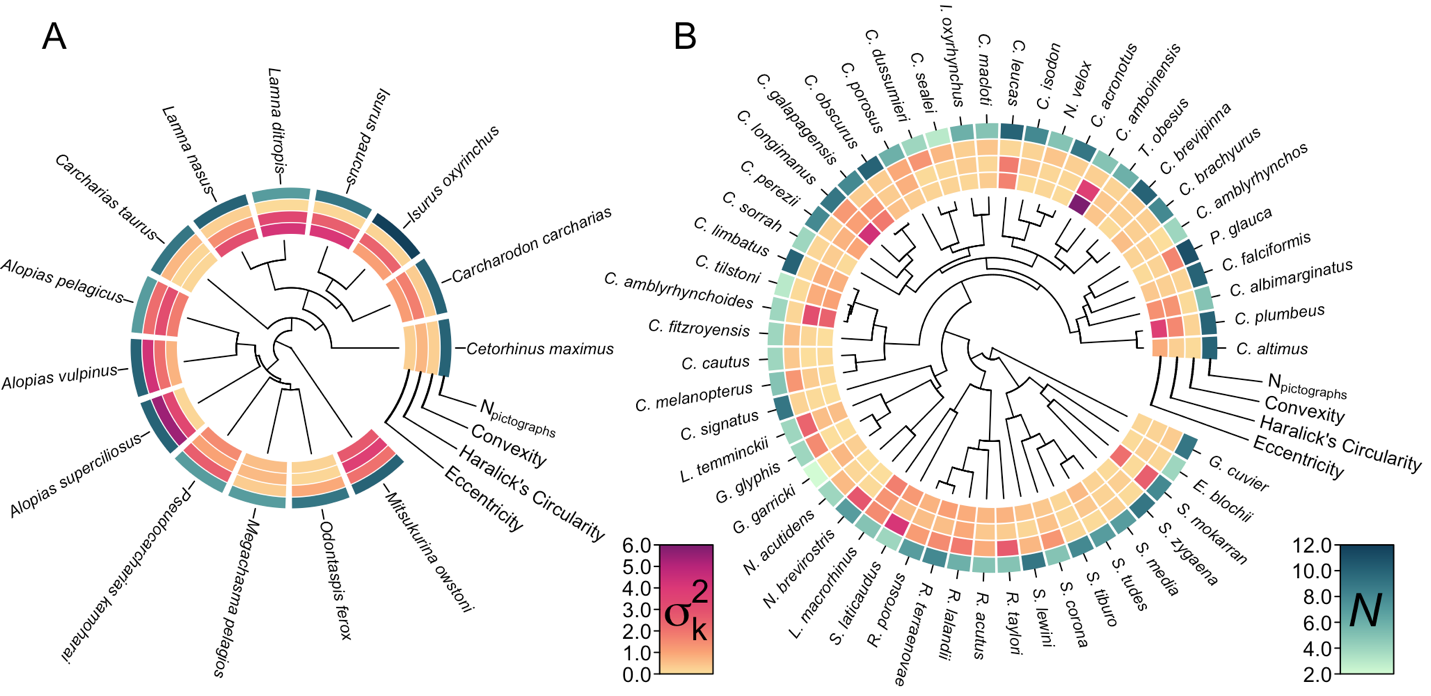


(A) The Lamniformes and (B) the Carcharhinidae + Sphyrnidae phylogenies with the scale parameter estimated by the GLMMs of eccentricity, Haralick’s circularity, and convexity global shape measurements $\left( \sigma_{k}^{2} \right)$ as well as the number of pictographs $\left( N_{\text{pictographs}} \right)$. Warmer colors indicate lower scale values (or less pictographs) while cooler colors indicate higher scale values (or more pictographs).

## Supplemental Figure 9 – Ancestral Trait Reconstruction

The node depth of the Lamniform phylogeny in millions of years as a function of the PC1_EF_ and PC2_EF_ score. For the tips (node depth at the minimum), PC scores are the mean across pictographs for the species while, for the internal nodes, the PC scores are from an ancestral trait reconstruction.

## Supplemental Figure 10 – Significant PCs in the PGLMM


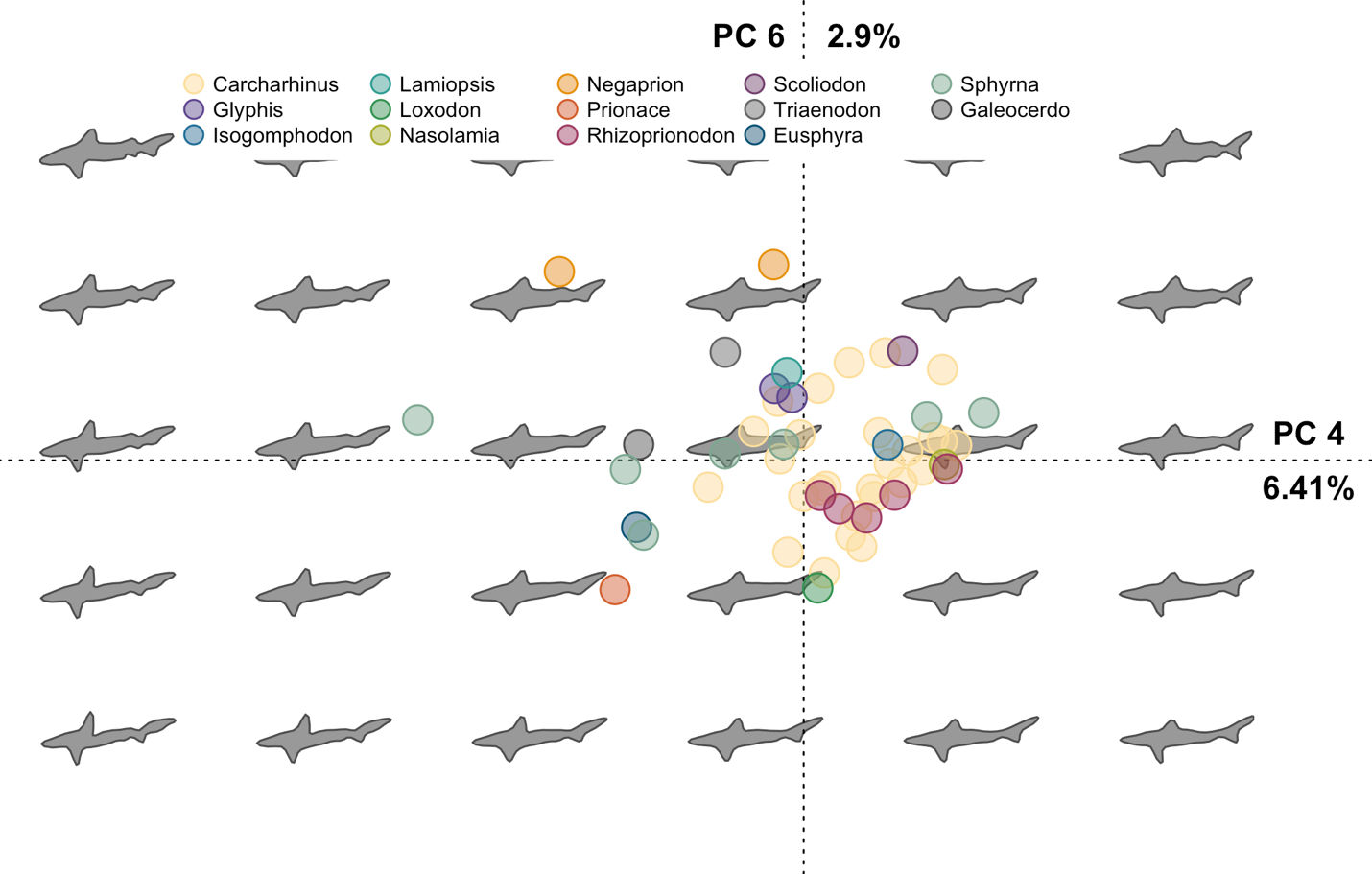


The fourth and sixth principal components (PC_EF_) of the Carcharhinidae and Sphyrnidae lateral profile pictographs. The expected body shape is indicated by the silhouettes while the mean PC_EF_ scores for the species are shown in dots and colored by genera. The percent variance explained of the two PC_EF_ axes is labeled on the x and y axes.

## Supplemental Figure 11 – PGLMM predictions

The observed and the predicted logit-transformed Endemism from the PC_EF_ model (A) and the PC_SL_ PGLMMs (B) for Carcharhinids and Sphyrnids. Circle corresponds to the sum of PC_EF_ scores (A) and PC_SL_ scores (B) from PC axes with significant effects in the model.

## Supplemental Figure 12 – Correlation between allometry measurements

Correlation between the eight proportion of total length measurements derived from the standardized length measurements. Warmer colors indicate positive correlation while cooler colors indicate negative correlations.
